# Surprisingly long lifetime of methacrolein oxide, an isoprene derived Criegee intermediate, under humid conditions

**DOI:** 10.1038/s42004-021-00451-z

**Published:** 2021-02-05

**Authors:** Yen-Hsiu Lin, Cangtao Yin, Kaito Takahashi, Jim Jr-Min Lin

**Affiliations:** 1 Institute of Atomic and Molecular Sciences, Academia Sinica, Taipei, Taiwan; 2 Department of Chemistry, National Taiwan University, Taipei, Taiwan

**Keywords:** Atmospheric chemistry, Reaction kinetics and dynamics

## Abstract

Ozonolysis of isoprene, the most abundant alkene, produces three distinct Criegee intermediates (CIs): CH_2_OO, methyl vinyl ketone oxide (MVKO) and methacrolein oxide (MACRO). The oxidation of SO_2_ by CIs is a potential source of H_2_SO_4_, an important precursor of aerosols. Here we investigated the UV-visible spectroscopy and reaction kinetics of thermalized MACRO. An extremely fast reaction of *anti*-MACRO with SO_2_ has been found, *k*_SO2_ = (1.5 ± 0.4) × 10^−10^ cm^3^ s^−1^ (±1*σ*, *σ* is the standard deviation of the data) at 298 K (150 − 500 Torr), which is ca. 4 times the value for *syn*-MVKO. However, the reaction of *anti*-MACRO with water vapor has been observed to be quite slow with an effective rate coefficient of (9 ± 5) × 10^−17^ cm^3^ s^−1^ (±1*σ*) at 298 K (300 to 500 Torr), which is smaller than current literature values by 1 or 2 orders of magnitude. Our results indicate that *anti*-MACRO has an atmospheric lifetime (best estimate ca. 18 ms at 298 K and RH = 70%) much longer than previously thought (ca. 0.3 or 3 ms), resulting in a much higher steady-state concentration. Owing to larger reaction rate coefficient, the impact of *anti*-MACRO on the oxidation of atmospheric SO_2_ would be substantial, even more than that of *syn*-MVKO.

## Abstract (web summary)

Methacrolein oxide is one of the ozonolysis products of isoprene present in our atmosphere, but its relevance under humid conditions is not fully understood. Here, UV-vis spectroscopy and reaction kinetics studies show much longer lifetimes of *anti*-methacrolein oxide than previously thought, suggesting much higher steady-state concentrations and higher impact on the oxidation of atmospheric SO_2_.

## Introduction

Isoprene is the most abundant unsaturated hydrocarbon in the atmosphere^1^. Ozonolysis of isoprene produces three kinds of carbonyl oxides (also called Criegee Intermediates, CIs): CH_2_OO, methyl vinyl ketone oxide (MVKO: CH_3_C(C_2_H_3_)OO), and methacrolein oxide (MACRO: CH_2_=C(CH_3_)CHOO)^2–4^. CIs are very reactive species. In 2012, their reactions with SO_2_ were found to be faster than previously thought by orders of magnitude^5^. The oxidation of SO_2_ (SO_2_ → SO_3_) has gained wide attention because it is an important process in the formation of secondary aerosols (SO_3_ → H_2_SO_4_)^6–15^. Field and chamber studies, pioneered by Mauldin et al.^9^, indicate that there is a non-OH oxidant contributing to the oxidation of atmospheric SO_2_ and this new oxidant may be Criegee intermediates^9–15^. However, it is impractical to measure the very reactive CIs in the atmosphere. Their atmospheric concentrations can only be estimated through kinetics analysis. For example, Novelli et al. have given an average estimate of the CI concentration of ca. 5 × 10^4^ molecules cm^−3^ (with an order of magnitude uncertainty) for the two environments they studied^16^. (For simplicity, we will use ‘cm^−3^’ for the unit of molecular number density, instead of the more formal ‘molecules cm^−3^’.) Note that older estimations may have larger uncertainties in the CI concentrations since the related reaction kinetics were not well determined at that time.

On the other hand, laboratory studies on individual CI reactions have revealed that the reactivity (thus the atmospheric fate) of a CI would strongly depend on its structure^17,18^. For CH_2_OO and *anti*-CH_3_CHOO, which have a H-atom at the *syn* position, the main decay pathway is their reactions with water vapor (H_2_O monomer and dimer)^18–23^. These reactions are extremely fast, resulting in very low steady-state concentrations of such CIs, which are too low to oxidize atmospheric SO_2_ at any substantial level^17^. For *syn*-CH_3_CHOO and (CH_3_)_2_COO, which have an alkyl group at the *syn* position, their unimolecular reactions via intramolecular 1,4-H-atom transfer are the major decay process, which also generates OH radicals^24–31^. These unimolecular processes are not slow and essentially limit the steady-state concentrations of such CIs^17,32^.

Different from alkyl-substituted CIs, MVKO and MACRO have a C=C double bond, which forms extended conjugation with the carbonyl oxide functional group (resonance stabilized). The pioneering works of Lester and coworkers have demonstrated a photolytic synthesis method that allows direct detection of MVKO and MACRO^33,34^. Via this new synthesis scheme, recent studies have shown that the resonance-stabilization would affect the reactivity and thus the atmospheric fate of MVKO^35,36^.

For MVKO, there are four possible isomers (or conformers)^2,33,35,37^. Similar to simpler CIs, the barrier of rotating the carbonyl C=O bond is high, resulting in non-interconverting *syn* and *anti* isomers (following the nomenclature of Barbar et al.)^33,38^. However, the barrier of rotating the C─C single bond between the C=C and C=O bonds is low, giving essentially an equilibrium mixture of *cis* and *trans* conformers^32,39,40^. It has been predicted that *anti*-*trans*-MVKO would quickly interconvert to *anti*-*cis*-MVKO (>10^6^ s^−1^)^32^, which decays quickly via fast 1,5-ring closure to form dioxole with a rate coefficient of ca. 2100 s^−1^ at 298 K^32,33,41,42^. As a result, *anti*-MVKO was not observed experimentally, presumably due to short lifetime and/or low yield^35^.

Caravan et al. have found that *syn*-MVKO reacts with SO_2_ and formic acid as fast as other alkyl CIs do. Furthermore, based on their global chemistry and transport model, they have shown that *syn*-MVKO could significantly increase the atmospheric oxidation of SO_2_ and the removal of formic acid, where the isoprene emission is high. The high impact of *syn*-MVKO is mostly due to the abundance of isoprene (its source) and its slow decay (slow unimolecular decay and slow reaction with water vapor)^35^. The slow decay of *syn*-MVKO is related to its resonance-stabilized electronic structure^32,36^, which would be disrupted at the transition state of the unimolecular reaction^33,43^. Another interesting aspect of this resonance stabilization is that the iodine-atom adduct of MVKO is relatively less stable compared to the cases of alkyl CIs^36^.

**Fig. 1:**
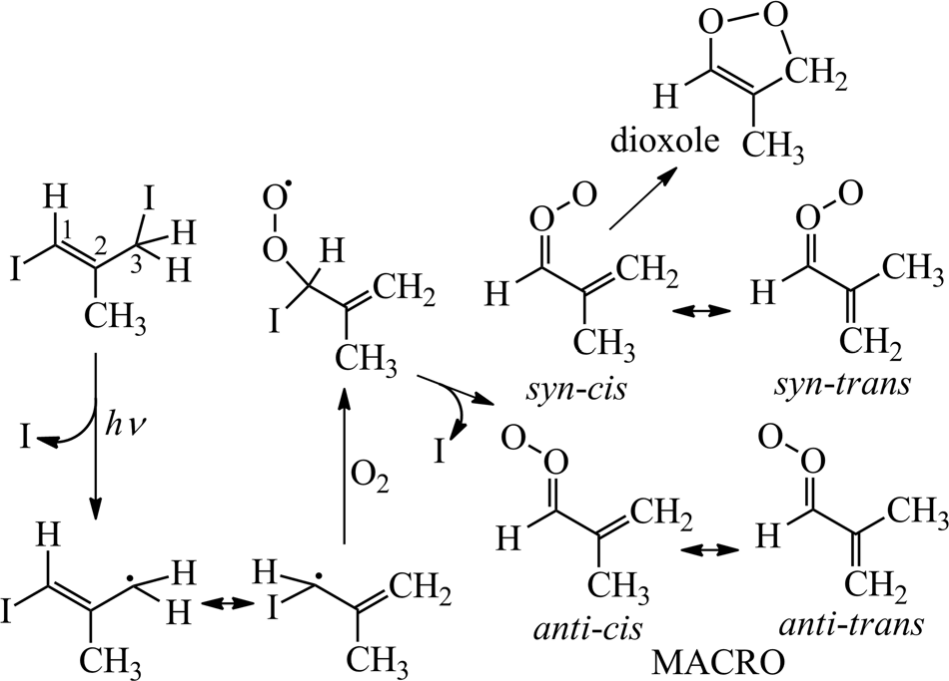
Synthesis and conformers of methacrolein oxide (MACRO). We follow the method and nomenclature of Vansco et al.^34^.

MACRO also has a resonance-stabilized electronic structure and two non-interconverting families of conformers (Fig. 1). It has also been predicted that *syn*-*trans*-MACRO (following the nomenclature of Vansco et al.)^34^ would interconvert quickly (>10^6^ s^−1^) to *syn*-*cis*-MACRO, which would undergo fast unimolecular decay (*k*_uni_ = 2500 s^−1^) to form dioxole, while *anti* conformers are expected to have slow unimolecular decay (ca. 10 s^−1^)^32^. These theoretically predicted values are from Vereecken et al.^32^ who utilized the structure–activity relationships which considered the best available theoretical and experimental results at that time. However, similar to other *anti* types of CIs (CH_2_OO and *anti*-CH_3_CHOO)^17,18^, the reaction of *anti*-MACRO with water vapor was predicted to be fast (*k*_water*-*eff_ = 7.2 × 10^−15^ cm^3^ s^−1^ by Anglada et al.^44^ or 6.3 × 10^−16^ cm^3^ s^−1^ by Vereecken et al.^32^ at relative humidity (RH) = 70% and 298 K, considering both water monomer and dimer reactions). If so, the fast reaction with water vapor (ca. 10^3^ s^−1^) would result in a very low steady-state concentration of *anti*-MACRO, diminishing its atmospheric impact. Nonetheless, as will be shown later, this picture is incorrect.

Very recently, Vansco et al. have reported the electronic spectroscopy and photochemistry of MACRO; as the authors have mentioned, “This UV–visible detection scheme will enable study of its unimolecular and bimolecular reactions under thermal conditions of relevance to the atmosphere.”^34^ Following their method, here we prepared MACRO starting from the photolysis of *E*-1,3-diiodo-2-methylprop-1-ene precursor (Fig. 1). The time-resolved UV–visible spectrum of MACRO was recorded by using a continuous broadband light source and a grating spectrometer equipped with an ultrafast CMOS camera. Analyzing the time series of the spectra allowed us to retrieve the spectrum of MACRO and its time-dependent concentration.

To our surprise, the reaction of MACRO with water vapor was found to be much slower than previous predictions^32,44^ by one or two orders of magnitude, implying much longer atmospheric lifetime (ca. 18 ms vs. 3 or 0.3 ms^32,44^) and higher steady-state concentrations for atmospheric MACRO. On the other hand, the resonance-stabilized MACRO still reacts extremely fast with SO_2_. Based on the results of a recent global chemistry and transportation model of MVKO^35^, our data suggest that the impact of MACRO on the oxidation of atmospheric SO_2_ would also be substantial.

## Results and discussion

### Analysis of the observed UV spectrum

**Fig. 2:**
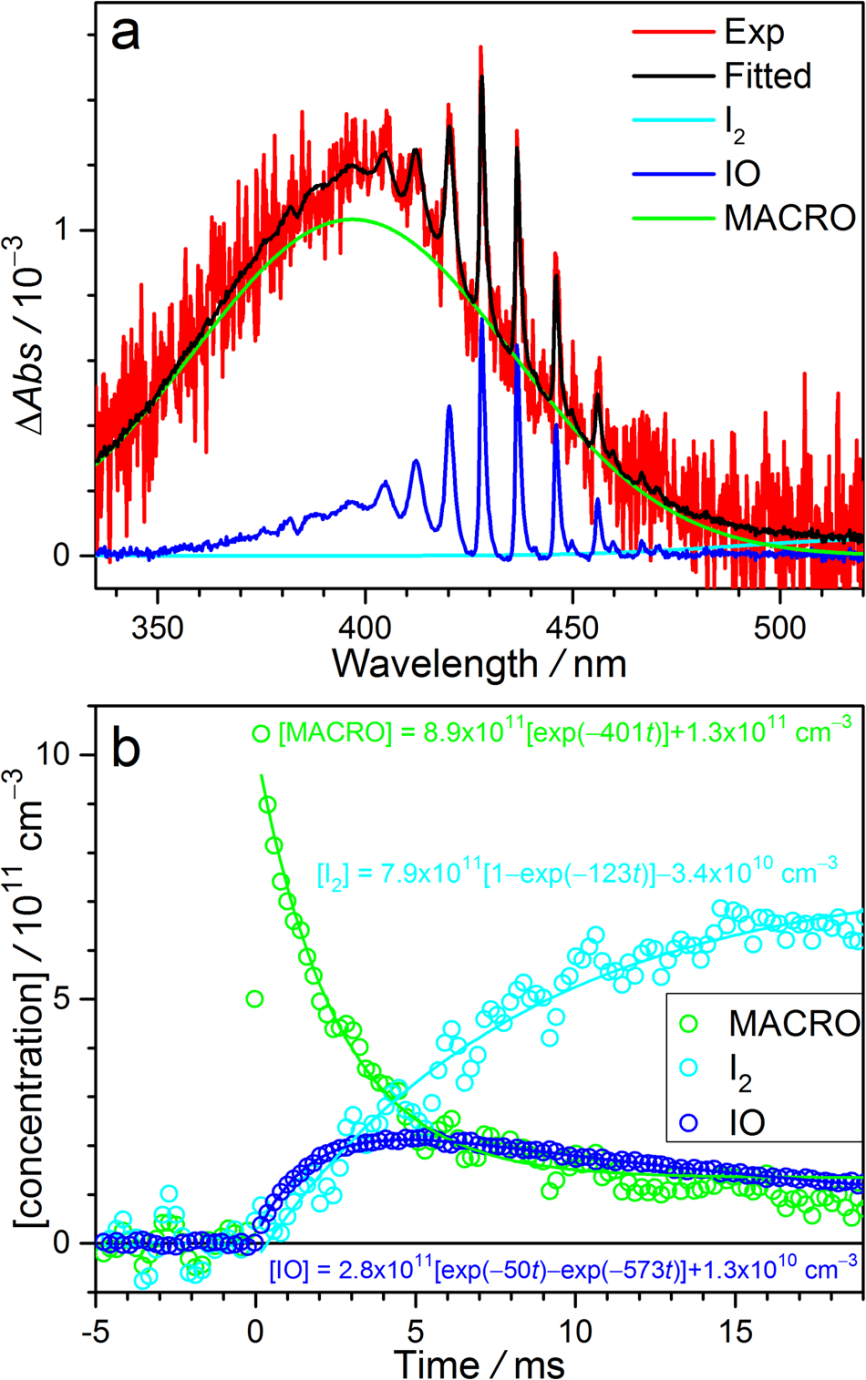
Absorption spectra and their time evolutions. **a** The difference absorption spectrum recorded in the 1,3-diiodo-2-methylprop-1-ene/O_2_ photolysis system at 298 K and 500 Torr at 0.4 ms photolysis-probe delay time (red line, the data is from Exp #13, see Supplementary Table 2). The absorption contributions of MACRO (green), IO (blue), and I_2_ (cyan; black is the sum of MACRO, IO, and I_2_) are also shown. **b** Concentrations of MACRO, IO, and I_2_ obtained by fitting the spectra of Exp #13 at [H_2_O] = 0 at each photolysis-probe delay time. [MACRO] is derived by using the reported peak cross section = 3 × 10^–18^ cm^2^ by Vansco et al.^34^, [IO] and [I_2_] are estimated with literature cross sections^45,46^ (see Supplementary Fig. 4).

Figure 2a shows the time-resolved difference absorption spectra recorded in the photolysis reactor. Here ‘difference’ means the change after the photolysis laser pulse. We can see three spectral features in the spectrum: (i) a very broad and structureless absorption band peaking at ca. 397 nm; (ii) absorption of IO which has distinctive sharp peaks between 400 and 460 nm^45^; (iii) broad absorption band of I_2_ extending to 520 nm^46^. The presence of IO and I_2_ is similar to previous investigations of CH_2_OO^47–49^, CH_3_CHOO^23,50^, (CH_3_)_2_COO^51^, and MVKO^35,36^.

### UV absorption spectrum of MACRO

Under the same experimental conditions, SO_2_ was added to scavenge CIs (see Supplementary Fig. 1). We found that the spectral feature (i) disappears, indicating its spectral carrier is a Criegee intermediate. We further subtracted the time-resolved spectra recorded at [SO_2_] = 1 × 10^14^ cm^−3^ from those without adding SO_2_ at each photolysis-probe delay time. This operation removed most of the absorption signals of IO and I_2_ (and also other minor byproducts), and the resulted spectra would be mainly from the Criegee intermediate. Considering that we were using the same precursor and preparation method of Vansco et al.^34^, we assigned this CI to MACRO.

**Fig. 3:**
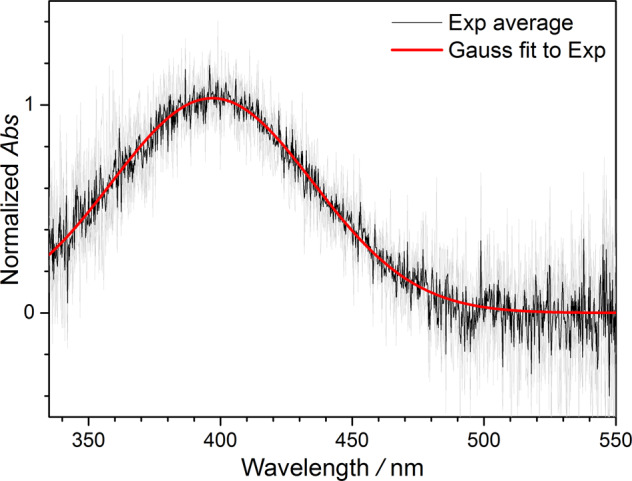
Height-normalized spectra of MACRO. The spectra of MACRO are obtained from the difference spectra between the spectra without SO_2_ and the spectra with [SO_2_] ≅ 10^14^ cm^−3^ at 0.18 ms delay time. Four spectra obtained from Exp #1−4 are plotted as gray lines, and their average is plotted as a black line. The red curve shows the Gaussian fit to the average spectrum with peak position 397 nm and full width at half-maximum (FWHM) 77 nm.

The spectrum of MACRO can be well fitted with a Gaussian function (Fig. 3). This spectrum is similar to that of Vansco et al. who reported a broad spectrum of MACRO peaked at 380 nm with weak oscillatory structure at long wavelengths ascribed to vibrational resonances^34^. However, we do not observe such oscillatory structure; the differences are presumably due to different temperatures of the MACRO samples (thus, the conformer populations may be different), as Vansco et al. recorded their spectrum under a jet cool condition^34^.

We may decompose the observed spectra into the contributions of MACRO, IO, and I_2_ (Fig. 2a and Supplementary Fig. 4) with a least-squares fit. The resulted signal intensities (converted to concentrations) of MACRO, IO, and I_2_ are plotted in Fig. 2b as a function of the delay time. The intensities of IO and I_2_ grow with time, indicating they are secondary products. While the kinetics of IO and I_2_ formation may be interesting, we like to focus on MACRO in this work. We can see that the lifetime of MACRO in this particular experiment is ca. 3 ms, much longer than the predicted value for *syn*-MACRO (<0.4 ms, based on its *k*_uni_ = 2500 s^−1^^32^; and other chemical processes would further shorten the lifetime). Therefore, we conclude that the observed spectral carrier should be *anti*-MACRO, similar to the case of MVKO^35^. Note that the long lifetime conformer of MVKO is *syn*-MVKO (following the nomenclature of Barbar et al.)^33^ which has a structure similar to *anti*-MACRO. For simplicity, we will use MACRO to represent *anti*-MACRO in the following analysis.

### Kinetics of MACRO reaction with SO_2_

**Fig. 4:**
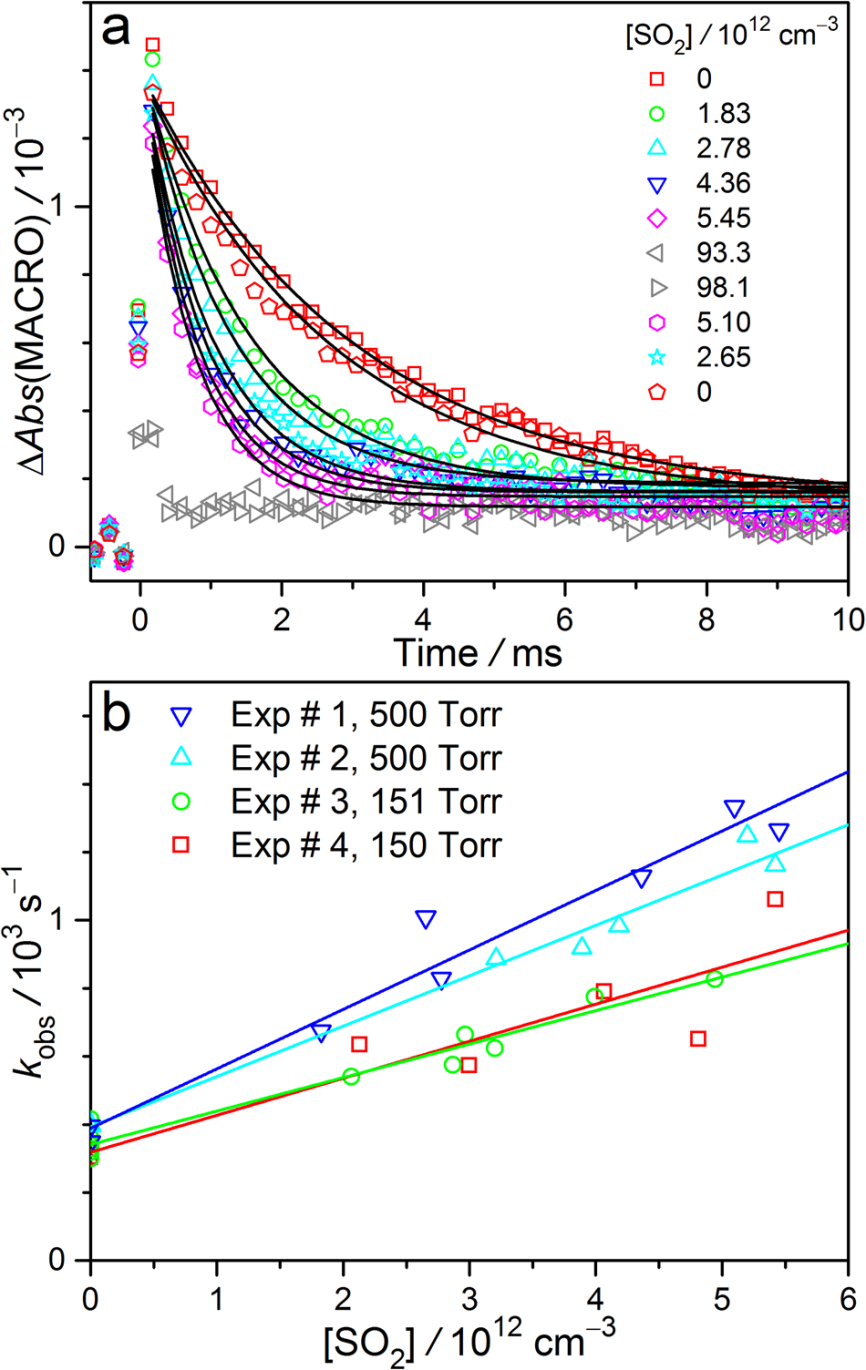
Kinetic plots of MACRO + SO_2_. **a** Time profiles of the absorbance changes of MACRO obtained by fitting the spectra at each delay time under various [SO_2_] at 298 K and 500 Torr. The data are from Exp #1. The black curves show the fitting results of single exponential decay. **b** Pseudo-first-order plot of the reaction rate coefficients of MACRO with SO_2_ at 298 K.

The above analysis has been repeated for experiments adding various [SO_2_]. The resulted MACRO signal intensities at each photolysis-probe delay time are plotted in Fig. 4a. The decay of MACRO signal can be fitted with a single exponential function to yield a pseudo-first-order rate coefficient, *k*_obs_, at each [SO_2_].

$${\Delta}Abs\left( {{\mathrm{MACRO}}} \right) = \sigma L\left[ {{\mathrm{MACRO}}} \right]\left( t \right) = \sigma L\left[ {{\mathrm{MACRO}}} \right]_0{\mathrm{exp}}\left( { - k_{{\mathrm{obs}}}t} \right)$$

where *σ* is the absorption cross section of MACRO^34^ and *L* is the optical path length (when reporting Δ*Abs*(MACRO), we use its peak value at 397 nm). Figure 4b shows that *k*_obs_ increases linearly with [SO_2_].

$$k_{{\mathrm{obs}}} = k_0 + k_{{\mathrm{SO}}2}\left[ {{\mathrm{SO}}_2} \right]$$

The slope would correspond to the rate coefficient *k*_SO2_ of the bimolecular reaction of MACRO with SO_2_, while the intercept *k*_0_ would account for other decay processes of MACRO that are independent on [SO_2_], like reactions with radical byproducts (including MACRO self-reaction), unimolecular decay, etc.

Because that MACRO can be fully scavenged within 0.3 ms under a high [SO_2_] (≥9.3 × 10^13^ cm^−3^), we may further improve the analysis by subtracting the high [SO_2_] spectrum (the average spectrum at the two highest [SO_2_]) from other low [SO_2_] spectra to remove most of the byproduct contributions while some minor amounts of IO and I_2_ may still remain (SO_2_ scavenge method). The resulted spectra were then decomposed into the contributions of MACRO and IO and I_2_. The time profiles of MACRO are plotted in Supplementary Fig. 2. When using this SO_2_ scavenge method, we did not include the data point at the first delay time (0.18 ms) due to incomplete scavenging.

The kinetic results of MACRO + SO_2_ reaction are summarized in Supplementary Table 1. The data at 150 and 500 Torr do not show significant difference after considering the experimental uncertainties. For these four sets of experimental data, we report the rate coefficient to be (1.5 ± 0.4) × 10^−10^ cm^3^ s^−1^ at 298 K and 150−500 Torr (±1*σ*, *σ* is the standard deviation of the data). The rate coefficients of SO_2_ reactions with other CIs (CH_2_OO^5,52^, *anti*- and *syn*-CH_3_CHOO^21,23^, (CH_3_)_2_COO^30,51^, MVKO^35,36^) are in the range of (0.4–2.2) × 10^−10^ cm^3^ s^−1^^17,18^. It appears that the resonance-stabilization of *anti*-MACRO does not reduce its reactivity towards SO_2_.

### Kinetics of MACRO reaction with H_2_O

**Fig. 5:**
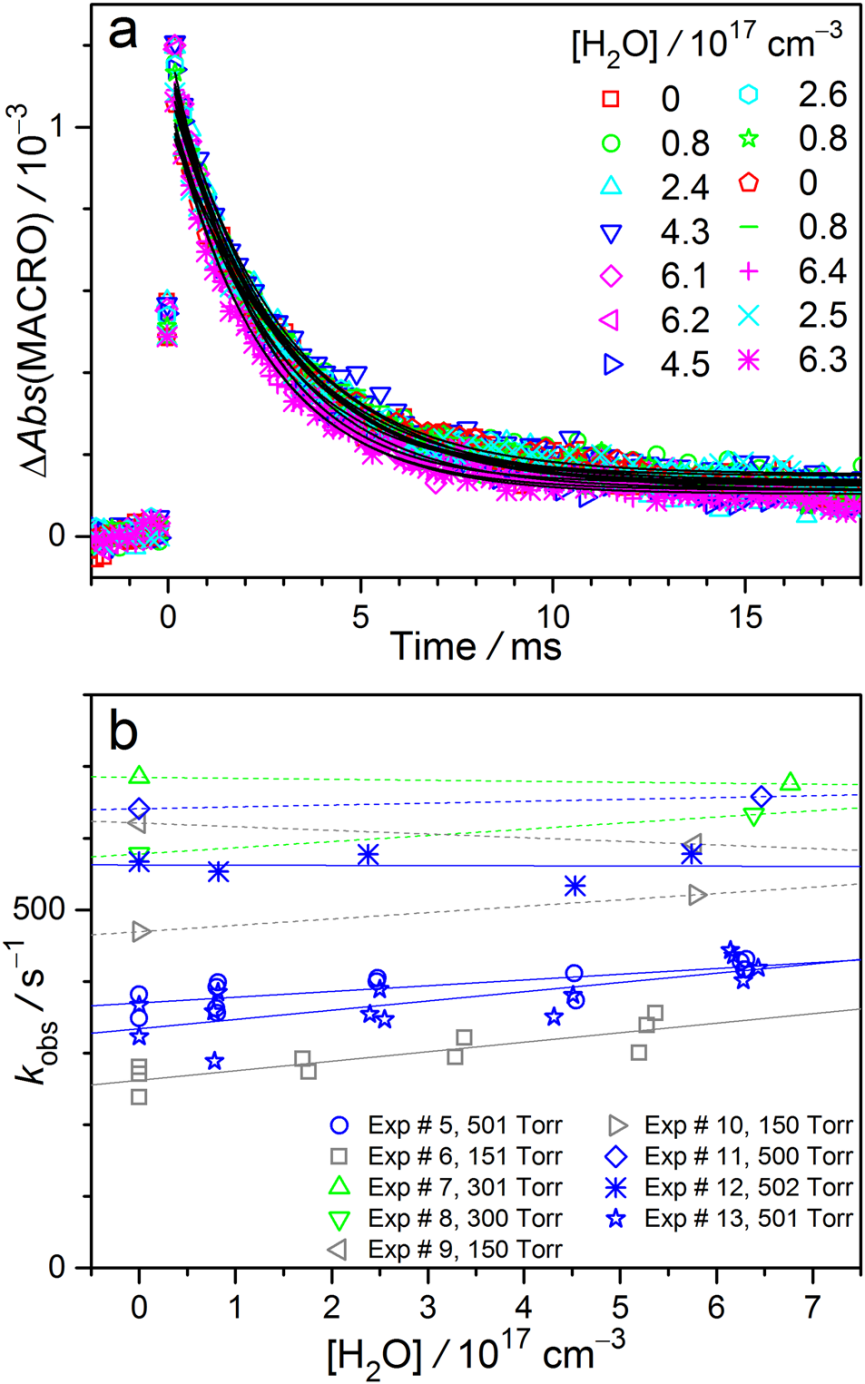
Kinetic plots of MACRO + H_2_O. **a** Time profiles of the MACRO absorption signal obtained by fitting the spectra at each delay time under various [H_2_O] at 298 K and 501 Torr. The data are from Exp #13 (Supplementary Table 2). The black curves show the fitting results of a single exponential decay function. **b** The pseudo-first-order plot of the reaction rate coefficients of MACRO with H_2_O at 298 K.

The same method has been applied to investigate the kinetics of MACRO reaction with water vapor. To our surprise, the effect of water in the decay of MACRO is rather weak as shown in Fig. 5, indicating slow reaction. From the plots of *k*_obs_ as a function of [H_2_O] (Fig. 5b), we can see that the slopes are quite insignificant; some of them are even negative, indicating that these rate measurements are close to our measurement limit (see Supplementary Table 2). Note that the highest [H_2_O] used is ca. 6 × 10^17^ cm^−3^ (ca. 18 Torr), which has replaced a larger portion (18/150 = 12%) of the bath gas if the total pressure is only 150 Torr (N_2_ balance). Thus, we think it may require some cautions to view the data of 150 Torr, because the reaction environment (type of bath gas) changes at various [H_2_O]. Nonetheless, no trend can be found for pressures from 150 to 500 Torr. Finally, we chose the weighted average from six experimental sets (300 and 500 Torr and 298 K, Supplementary Table 2), to report the effective rate coefficient for the reaction of MACRO with water vapor, *k*_water*-*eff_ = (9 ± 5) × 10^−17^ cm^3^ s^−1^ (±1*σ*). As mentioned above, we are not confident enough to determine the lower limit of *k*_water*-*eff_.

### MACRO isomers

As pointed out by Vereecken et al., the *cis*–*trans* interconversion (Supplementary Fig. 6) (>10^6^ s^–1^) is orders-of-magnitude faster than other chemical processes, such that the *cis* and *trans* conformers will be in near-equilibrium and should be considered as a single pool of species^32^. Following this idea, we summarize the rate coefficients of the unimolecular processes and reactions with water vapor (monomer and dimer^53^) in Table 1 for relevant CIs^32,35,43,44^.

**Table 1:** Summary of theoretically estimated unimolecular rate coefficient *k*_uni_ and bimolecular rate coefficients of the reactions with water monomer and dimer (*k*_H2O_ and *k*_(H2O)2_) at 298 K for selected CIs.

Relative humidity				70%		35%		
[H_2_O]/10^17^ cm^−3^				5.4		2.7		
[(H_2_O)_2_]/10^14^ cm^−3^				6.7		1.7		
CI	*k*_uni_ (s^−1^)	*k*_H2O_ (cm^3^ s^−1^)	*k*_(H2O)2_ (cm^3^ s^−1^)	*k*_water-eff_^a^ (cm^3^ s^−1^)	*k*_atm_^b^ (s^−1^)	*k*_water-eff_^a^ (cm^3^ s^−1^)	*k*_atm_^b^ (s^−1^)	Ref.
*syn*-MVKO	33	3.4 × 10^−19^	9.2 × 10^−16^	1.5 × 10^−18^	34	9.1 × 10^−19^	33	^35,44^
	50	8.1 × 10^−20^	3.1 × 10^−16^	4.6 × 10^−19^	50	2.7 × 10^−19^	50	^32^
	[70 ± 15]	–	–	−	70	−	70	^43^
*anti*-MACRO	–	3.0 × 10^−15^	3.4 × 10^−12^	7.2 × 10^−15^	3900	5.1 × 10^−15^	1400	^44^
	10	1.9 × 10^−16^	3.6 × 10^−13^	6.3 × 10^−16^	350	4.1 × 10^−16^	120	^32^
	< 25	4.9 × 10^−17^	1.6 × 10^−14^	6.9 × 10^−17^	< 62	5.9 × 10^−17^	< 41	Theory^c^
	7^d^		–	[(9 ± 5) × 10^−17^]	56	[(9 ± 5) × 10^−17^]	31	This work
*syn*-MACRO	−	2.3 × 10^−19^	2.4 × 10^−15^	1.3 × 10^−17^	–	1.7 × 10^−18^	−	^44^
	2500	1.5 × 10^−19^	5.6 × 10^−16^	8.4 × 10^−19^	2500	5.0 × 10^−19^	2500	^32^
	2600	1.4 × 10^−20^	4.1 × 10^−17^	6.4 × 10^−20^	2600	3.9 × 10^−20^	2600	Theory^c^

Available experimental results are shown in square brackets.

^a^*k*_water-eff_ = (*k*_H2O_[H_2_O] + *k*_(H2O)2_[(H_2_O)_2_])/[H_2_O] at 298 K, in which [(H_2_O)_2_] is estimated with *K*_eq_ = *P*_dimer_/*P*_monomer_^2^ = 0.0556 bar^−1^ reported by Anglada et al.^53^.

^b^*k*_atm_ = *k*_uni_ + *k*_water-eff_[H_2_O].

^c^This work, based on QCISD(T)/CBS//B3LYP/6-311+G(2*d*,2*p*) (CBS = complete basis set extrapolated).

^d^Our best estimated theoretical value (see text).

As shown in Table 1, the predicted unimolecular decay rates are very different for *syn*- and *anti*-MACRO. *Syn*-MACRO would have a rather short lifetime of ca. 1/2500 = 4 × 10^−4^ s^32^, which means its steady-state concentration would be very low. The experimental lifetime of MACRO is found to depend on the signal intensity—the higher the signal is, the shorter the lifetime. This is due to the fact that the inevitable reactions of MACRO with radical byproducts, including I atoms, IO radicals, MACRO itself and the products from the fast decomposition of *syn*-MACRO (similar to the case of *anti*-MVKO)^42^ would shorten its lifetime. Supplementary Fig. 3 shows the plot of *k*_obs_ against [MACRO]_0_. The linear relationship supports the above mechanism. Extrapolating *k*_obs_ to zero [MACRO]_0_ would effectively remove the bimolecular contributions and give an estimate for the unimolecular lifetime of MACRO. The preliminary data of Supplementary Fig. 3 are consistent with *k*_uni_ ≅ *k*_obs_([MACRO]_0_ = 0) < 50 s^−1^, which gives a lifetime > 20 ms. This long-lived MACRO cannot be *syn*-MACRO. Thus, the observed signal should belong to *anti*-MACRO.

### Compare of *k*_water-eff_ with previous theory

The value of *k*_water*-*eff_ of *anti*-MACRO (Table 1) is smaller than those of CH_2_OO and *anti*-CH_3_CHOO^17,18^ by orders of magnitude, suggesting that the extended conjugation of *anti*-MACRO correlates with the lower reactivity towards water vapor (monomer and dimer), since the alkyl-substituted CIs lack the resonance-stabilized electronic structure of the extended conjugation.

Anglada et al. have predicted the rate coefficients for *anti*-MACRO reactions with water monomer and dimer, giving *k*_water-eff_ = 7.2 × 10^−15^ cm^3^ s^−1^ at RH = 70% and 298 K (Table 1)^44^. To our surprise, this value is ca. 80 times larger than our experimental value. However, Vereecken et al. have pointed out that the level of theory used by Anglada et al.^44^ tends to underestimate the barriers for the CI reactions with water monomer and dimer^32^. Using ‘structure–activity relationship’, Vereecken et al. scaled the barrier heights of a number of CI reactions by considering the best known theoretical and experimental data (mainly for CH_2_OO and *anti*-/*syn*-CH_3_CHOO) at that time^32^. The resulted rate coefficients of MACRO are also shown in Table 1. We can see this ʻscalingʼ does reduce the gap (from 80 times to 7 times) between the theoretical predictions and our experimental data. Since the reference data in the work of Vereecken et al.^32^ do not contain trustable data for reactions of CIs having a conjugated C=C group (i.e., there is no good anchor point for the scaling), this difference may be reasonable. Also note that Vereecken et al.^32^ have estimated an uncertainty of one order of magnitude for their rate coefficients at 298 K.

With details given in Supplementary Note 3, we found that it is important to calculate the reaction barrier heights at a high level of quantum chemistry theory like QCISD(T)/CBS//B3LYP/6-311+G(2*d*,2*p*) (CBS = complete basis set extrapolation)^54–60^. For example, the QCISD(T)/CBS barriers are ca. 1.4 or 2.0 kcal mol^−1^ higher than those calculated at QCISD(T)/aug-cc-pVTZ (AVTZ) for various reactions between (CH_2_=CH)CHOO conformers with H_2_O monomer or dimer (Supplementary Fig. 7 and Supplementary Table 4), indicating that only using the AVTZ barrier heights would overestimate the reaction rates significantly.

After properly scaling the effect of the basis sets (CBS vs. AVTZ) by using the results of (CH_2_=CH)CHOO, which has a structure similar to MACRO, as an anchor point, (Supplementary Fig. 7)^54^, our calculation (Table 1) also predicts slower rates compared to previous ones.

### Compare of *k*_water-eff_ with ozonolysis experiment

Newland et al. have analyzed the effect of water vapor in the system of isoprene ozonolysis; in their two-CI model, the isoprene-derived non-CH_2_OO CI (sum of MVKO and MACRO) has an effective reaction rate coefficient with water vapor of (1.1 ± 0.27) × 10^−15^ cm^3^ s^−1^^61^. While all conformers of MVKO are expected to react with water vapor much slower (*k*_water-eff_ ≤ 10^−17^ cm^3^ s^−1^)^44^, the value of Newland et al. is much larger than our *k*_water-eff_ for MACRO. At the time (2015) when the work of Newland et al. was published, the knowledge of the reaction kinetics of MVKO and MACRO was not available at all. As multiple CIs are produced in the isoprene ozonolysis system, the kinetics is rather complicated, especially when these CIs have very different reactivities towards water vapor. For example, CH_2_OO, which has the predominant yield in the isoprene ozonolysis system^2,3^, would be quickly consumed by its reaction with water vapor, but MVKO and MACRO would not. See Supplementary Note 2 for an alternative analysis to fit the data of Newland et al.^61^. In fact, Newland et al. have mentioned that the competing effects of the different kinetics of two distinct forms (*syn* and *anti* conformers) in the system may effectively lead to one masking the other under the experimental conditions applied^61^.

### Best estimation of *k*_uni_

It is very difficult to experimentally measure the very slow rate of the *anti*-MACRO unimolecular reaction. While our preliminary experimental data (Supplementary Fig. 3) suggest that the unimolecular reaction is slow, we cannot nail down the value of *k*_uni_ by the experimental results. On the theoretical side, the unimolecular reaction of *anti*-MACRO proceeds through the OO bending channel forming dioxirane^32,34,39^, similar to that of CH_2_OO^41,62^. By comparing with the results of high-accuracy extrapolation protocols like HEAT-345(Q)^62^ or high-level multireference methods like MRCI+Q (Davidson correction)/CBS^41^, Yin and Takahashi have found that the QCISD(T)/CBS method slightly underestimates the barrier height of this channel (by ca. 0.4 or 1.2 kcal mol^–1^, respectively) for CH_2_OO^41^. Our analysis in Supplementary Note 3 shows that for the MACRO unimolecular reaction, the electronic energy obtained by QCISD(T)/CBS would consistently underestimate the barrier height and other factors in the rate calculation, like hindered-rotor partition function calculation and tunneling correction, have very minor effects compared to that of the electronic energy. Therefore, our theoretical value (25 s^−1^) of *k*_uni_ of *anti*-MACRO would only represent an upper limit.

Assuming such underestimation in the barrier heights (0.4 or 1.2 kcal mol^–1^) is similar for the unimolecular reactions of MACRO and CH_2_OO, we may have an overestimation of a factor of 2 or 7 for the reaction rate coefficient at 298 K. Thus, we think the best estimated *k*_uni_ at 298 K would be ca. 25/(2 × 7)^0.5^ = 7 s^−1^ (the uncertainty may be up to a factor of 3), which is (almost) coincident with the theoretical value of 10 s^−1^ by Vereecken et al. (claimed uncertainty is ca. a factor of 5 for non-H-migration reactions)^32^. Although the uncertainty is still not very small, “for many assessments, however, it is sufficient to determine whether the reaction is significantly faster or slower than competing reactions”, mentioned by Vereecken et al.^32^.

### Atmospheric lifetime

Because the unimolecular decay and reaction with water vapor are the predominant processes that determine the atmospheric lifetime of a CI^17,18,32^, we may estimate the effective decay rate coefficient *k*_atm_ for atmospheric *anti*-MACRO.

$$k_{{\mathrm{atm}}} = k_{{\mathrm{uni}}} + k_{{\mathrm{water}} - {\mathrm{eff}}}\left[ {{\mathrm{H}}_2{\mathrm{O}}} \right]$$

Taking the best estimated *k*_uni_ (7 s^−1^) and our experimental data of *k*_water-eff_ (Table 1), we have *k*_atm_ = 56 s^−1^ (or <74 s^−1^, if taking our theoretical upper limit of 25 s^−1^ for *k*_uni_) for *anti*-MACRO at RH = 70% and 298 K. Note that the water reaction may still predominate in the decay processes of atmospheric *anti*-MACRO under humid conditions that are typical for tropical forests where the isoprene emission is large. And this atmospheric lifetime (ca. 18 ms, best estimate) is much longer than previously thought (0.3 or 3 ms, see Table 1), indicating that the atmospheric concentration of *anti*-MACRO would be much higher than previously expected. If using the upper limits of *k*_uni_ (25 s^−1^) and *k*_water-eff_[H_2_O] (49 + 54 = 103 s^−1^, 2*σ* upper bound, at RH = 70% and 298 K), we then have *k*_atm_ < 128 s^−1^, which would correspond to a lifetime longer than 7.8 ms.

### Impact of *anti*-MACRO on the oxidation of atmospheric SO_2_

This would depend on three factors: (i) the yield of *anti*-MACRO in the ozonolysis of atmospheric alkenes (mainly isoprene), (ii) the atmospheric lifetime of *anti*-MACRO, and (iii) the rate coefficient of *anti*-MACRO reaction with SO_2_. Each factor is discussed below.

First, based on the recent analysis of Nguyen et al., *anti*-MACRO has a yield of 15% among all stabilized CIs in isoprene ozonolysis, which is very similar to that of *syn*-MVKO (14%)^2,17^. In addition, an earlier study of Zhang and Zhang has shown that the activation energies of O_3_ cycloaddition to the two double bonds of isoprene are comparable and the barrier heights from the primary ozonides to *syn*-MVKO and *anti*-MACRO are also similar, implying that the *syn*-MVKO and *anti*-MACRO pathways are both accessible^4^.

Second, given that *syn*-MACRO and *anti*-MVKO have much shorter lifetimes (*τ* <1 ms), the oxidation of atmospheric SO_2_ by the C4 CIs from isoprene ozonolysis would be mainly by *anti*-MACRO and *syn*-MVKO (*τ* > 10 ms)^32,35^. The order of magnitude of *k*_atm_ of *anti*-MACRO (56 s^−1^) is comparable to that of *syn*-MVKO (*k*_atm_ ≅ *k*_uni_ = 33 s^−1^ or 70 s^−1^^33,43^; *k*_water-eff_ ~ 10^−18^ cm^3^ s^−1^)^32,35^. Combined with their similar yields in the isoprene ozonolysis, this suggests that *anti*-MACRO and *syn*-MVKO would have similar steady-state concentrations ([*anti*-MACRO]_ss_ ≈ [*syn*-MVKO]_ss_) in the troposphere.

Finally, the rate coefficient of SO_2_ reaction with *anti*-MACRO, (1.5 ± 0.4) × 10^−10^ cm^3^ s^−1^, is larger than that with *syn*-MVKO, (4.0−4.2) × 10^−11^ cm^3^ s^−1^^35,36^, by a factor of ca. 4. Overall, the oxidation rate of SO_2_ by *anti*-MACRO would be larger than that by *syn*-MVKO by a factor of ([*anti*-MACRO]_ss_/[*syn*-MVKO]_ss_)(*k*_SO2_*anti*-MACRO_/*k*_SO2_*syn*-MVKO_). This factor would be larger than unity, if we assume that these two CIs are mainly from isoprene ozonolysis with similar yields.

Although CH_2_OO has the highest yield (ca. 58%) among the stabilized CIs produced in the ozonolysis of isoprene^2,17^, its fast reaction with water vapor results in a rather short atmospheric lifetime (<1 ms)^17–20^, too short for CH_2_OO to reach any substantial concentration to oxidize atmospheric SO_2_. Recently Caravan et al., who utilized the up-to-date data of MVKO kinetics, show that *syn*-MVKO has the largest modeled steady-state concentration among all stabilized CIs globally (33% by molecules, 49% by weight)^35^. The above analysis shows that *anti*-MACRO would have similar concentrations as those of *syn*-MVKO and an even larger impact on the SO_2_ oxidation.

## Conclusion

Following the method of Vansco et al.^34^, MACRO has been synthesized and its UV–visible spectroscopy and reaction kinetics have been investigated. Similar to MVKO, MACRO has two non-interconverting isomers, *syn* and *anti* forms. *Syn*-MACRO would undergo fast 1,5-ring closure with a predicted thermal lifetime of <0.4 ms. In our experiments, a much longer lifetime (*τ* > 4 ms) has been observed, indicating that the spectral carrier is *anti*-MACRO. The rate coefficient of *anti*-MACRO reaction with SO_2_ has been determined to be (1.5 ± 0.4) × 10^−10^ cm^3^ s^−1^ at 298 K, which is substantially larger than that of the *syn*-MVKO + SO_2_ reaction. However, the reaction of *anti*-MACRO with H_2_O was found to be quite slow with an effective rate coefficient of (9 ± 5) × 10^−17^ cm^3^ s^−1^ at 298 K, which is smaller than previous theoretical values by 1 or 2 orders of magnitude. Theoretical calculations that properly treat the effect of the conjugated C=C substitution may reproduce the experimental trend.

A recent global chemistry and transport modeling based on the most up-to-date knowledge of MVKO chemistry has shown that *syn*-MVKO is important in the tropospheric processes of SO_2_ oxidation and formic acid removal^35^. Our results indicate that *anti*-MACRO has an atmospheric lifetime similar to that of *syn*-MVKO, resulting in a similarly substantial steady-state concentration. Combined with the larger rate coefficient of its reaction with SO_2_, the impact of *anti*-MACRO on the oxidation of atmospheric SO_2_ would be larger than (at least comparable to) that of *syn*-MVKO.

As mentioned above, to serve as an efficient oxidant of SO_2_, it is required to have a long-enough lifetime under atmospherically relevant conditions. As shown above and in the literature^35^, a resonance-stabilized electronic structure plays an interesting role for CIs. It reduces the reactivity for unimolecular decay and reactions with water vapor, but not for the reactions with SO_2_. Thus, having a resonance-stabilized electronic structure may be a new direction for searching for a long-lived CI that is able to oxidize atmospheric SO_2_.

## Methods

### MACRO preparation

The experimental setup has been published^19,36^. We prepared MACRO following Vansco et al.: ICHC(CH_3_)CH_2_I (1,3-diiodo-2-methylprop-1-ene, Accela, 97.8% by gas chromatography) + *hν* (248 nm) → CH_2_=C(CH_3_)CHI + I, CH_2_=C(CH_3_)CHI + O_2_ → CH_2_=C(CH_3_)CHOO + I (Fig. 1)^34^. The precursor concentrations were determined by its UV absorption spectra; the absolute cross sections (Supplementary Fig. 4) have been determined by measuring the weight loss of the precursor sample and the volume flow rate of the dilution gas^63,64^ (see Supplementary Note 1).

### CMOS camera spectrometer

A grating spectrometer (Andor SR303i) and fast CMOS camera (Andor, Marana-4BU11) were used to obtain the time-resolved absorption spectra of the reaction system. A series of spectra (exposure time 0.21 ms (or 0.43 ms) each) were recorded for every photolysis event. The spectrum taken before the photolysis laser pulse was used as the reference spectrum; therefore, the change of absorbance caused by the photolysis laser pulse was recorded transiently. Accumulation of 256, 512 (0.43 ms exposure time), or 1280, 2560 (0.21 ms exposure time) laser pulses was performed to improve the signal-to-noise ratio.

A background spectrum (without adding the MACRO precursor 1,3-diiodo-2-methylprop-1-ene) was recorded under the same experimental condition. This background was due to the interaction between the photolysis laser beam and the used optics (mainly the long-pass filters that reflected the photolysis laser beam and transmitted the probe beam). All the reported spectra are background corrected.

### Theoretical calculations

We optimized the reactant and transition state geometries on the singlet ground electronic state using B3LYP/6-311+G(2*d*,2*p*)^54,58,59^. See Supplementary Data 1 for the optimized XYZ geometries. The electronic energies were corrected at QCISD(T)/CBS level^54–60^, except for the transition states of MACRO + 2H_2_O, of which the energies were estimated with a correction method detailed in Supplementary Note 3 (Supplementary Figs. 7–9, Supplementary Tables 4–6). The rate coefficients were calculated using the conventional transition state theory method using rigid rotor harmonic oscillator approximations including tunneling correction.

## Supplementary information

Supplementary Information

Description of Additional Supplementary Files

Supplementary Data 1

Peer Review File

## Data Availability

The data supporting the findings of this study are available within the article, its Supplementary Information, and Supplementary Data 1 (XYZ geometries), and from the corresponding author upon reasonable request.
