# Supplementary material for: Surprisingly long lifetime of methacrolein oxide, an isoprene derived Criegee intermediate, under humid conditions
Source: Commun Chem. 2021 Feb 5;4:12. doi: 10.1038/s42004-021-00451-z (PMC9814537; doi:10.1038/s42004-021-00451-z)
Supplement: Supplementary file 1 — Supplementary Information [file 42004_2021_451_MOESM1_ESM.pdf]

Supplementary Information for

Surprisingly long lifetime of methacrolein oxide, an isoprene derived Criegee intermediate, under humid conditions

Yen-Hsiu Lin,[1] *Cangtao Yin,*[1] *Kaito Takahashi,*[1] *and Jim Jr-Min Lin.*[1,2] ✉

[1] Institute of Atomic and Molecular Sciences, Academia Sinica, Taipei 10617, Taiwan

[2] Department of Chemistry, National Taiwan University, Taipei 10617, Taiwan.

✉ Email: jimlin@gate.sinica.edu.tw

Table of Contents

Supplementary Note 1: Experimental S2

Experimental Supplementary Tables 1-2 S4

Experimental Supplementary Figures 1-4 S6

Supplementary Note 2: Alternative simulation for Figure 4 of Newland et al. S10

Supplementary Note 3: Theoretical calculations S14

References S27

Supplementary Note 1: Experimental

The details of our experimental system have been described elsewhere.[1,2] We prepared MACRO following the method reported by Vansco et al.: $ICHC(CH_3)CH_2I$ (1,3-diiodo-2-methylprop-1-ene) + $h\nu \rightarrow CH_2{=}C(CH_3)CHI$ + I, $CH_2{=}C(CH_3)CHI + O_2 \rightarrow CH_2{=}C(CH_3)CHOO$ + I.[3] The 248 nm excimer (Coherent, CompExPro 205) was used as the photolysis light source, and coupled into and out of the reactor by two long-pass filters (Asahi Spectra Co., Ltd. UV 275 nm, 25 mm dia. F0201 & F0204). The laser power was measured by an energy meter (Gentec EO, UP25N-100H-H9-D0) after reflecting out of the reactor. Our reactor was a glass tube (80 cm long, 1.9 cm inner diameter) with anti-reflection SiO_2 windows (Edmunds, 65875). The windows were purged by N_2 flows to prevent contamination, and the effective sample length was thus reduced to 71 cm. The flow of the precursor $ICHC(CH_3)CH_2I/N_2/O_2$ was mixed with the flow of the reactant (SO_2 or H_2O)/N_2 right before entering the reactor. All gas flows were controlled by mass flow controllers (Brooks, 5850E and Bronkhorst FG-201 CV). The total gas flow was set to fully refresh the reactor between consecutive laser pulses (laser repetition rate: 0.8–2.2 Hz). The probe light was from a broadband Xe lamp (Energetiq, EQ-99), and reflected six times through the reactor by a prism (Thorlabs, PS609, fused silica, right-angle, uncoated, 5 mm) and a spherical mirror (R = 1 m, Thorlabs, CM750-500-F01), extending the effective light path to 426 cm. The temperature of the reactor was 298.7±0.4 K. Another similar reactor, which was only 5 cm shorter, was used for some experiments.

[SO_2] and [$ICHC(CH_3)CH_2I$] measurements

The concentrations of the precursor, (*E*)-1,3-diiodo-2-methylprop-1-ene, ($ICHC(CH_3)CH_2I$, Accela, 97.8 % by gas chromatography) and SO_2 (Matheson, 99.98 %) were measured separately in two glass cells (length = 90 cm and inner diameter = 1.2 cm for both) by mini-spectrometers (Ocean Optics, FLAME-S-XR1-ES for $ICHC(CH_3)CH_2I$; Ibsen FHW-380 for SO_2) before entering the reactor, and derived by using the Beer-Lambert law.

$$Abs = \ln(I_0/I) = n\sigma L$$

Where Abs, I_0, I, n, σ, L are the absorbance, input light intensity, transmitted light intensity, molecular number density, absorption cross section, and optical path length, respectively.

We diluted SO_2 in N_2 in a 2.6-liter stainless steel cylinder before the experiments, and [SO_2] was derived by comparing the integration of SO_2 absorbance and that of the reference

cross section[4] within 200–350 nm.

The absolute absorption cross sections of the precursor were determined by recording the absorption spectra, the volume flow rate of the dilution gas (N_2), and the weight loss of the precursor sample, as in our previous works.[5,6] The resulted absolute cross sections are plotted in Supplementary Figure 4.

Concentration of H_2O

The concentration of H_2O vapor (vaporized from Milli-Q water, 18 MΩ) was controlled by the flow ratio of dry N_2 and moisturized N_2. The temperature and humidity of the mixture gas was measured by a precision humidity-temperature probe (Rotronic, HC2-S). The pressures at relevant locations were measured by diaphragm pressure gauges (INFICON, CDG025D 1000 Torr). The concentrations of the precursor and the reactants (SO_2 or H_2O) are derived by considering the flow, pressure, and temperature variations assuming the ideal gas behavior.

CMOS camera spectrometer

A grating spectrometer (Andor SR303i) and CMOS camera (Andor, Marana-4BU11) were used to obtain the time-resolved spectra of the reaction system. A series of spectra (exposure time 0.21 ms (or 0.43 ms) each) was recorded with each photolysis laser pulse. The spectrum taken before the photolysis laser pulse was used as a reference spectrum; therefore, the change of absorbance caused by the photolysis laser pulse was recorded transiently. A spectrum with exposure time 0.21 ms each was obtained by accumulating 2560 or 1280 laser pulses while that with exposure time 0.43 ms each was obtained by accumulating 256 or 512 laser pulses.

A background spectrum caused by the photolysis laser pulse and the long-pass filters that reflected the photolysis laser (other used optics might also contribute) was recorded in the experiments and all reported spectra have subtracted out the background spectrum.

Supplementary Table 1. Summary of the experimental conditions and fitting results of the reaction of MACRO with SO_2 at 298 K. k_{SO2} = (1.5±0.4)x10^{-10} cm^3 s^{-1} (±1 *sd*, *sd* = standard deviation of the data, considering all 8 data points from the two methods and 20% error in [SO_2]).

					Regular method[a]			SO_2 scavenge method[b]		
Exp #	P / Torr	P_{O2} / Torr	[precursor] / 10^{13} cm^{-3}	I_{248nm} / mJ cm^{-2}	[MACRO]$_0$ / 10^{11} cm^{-3}	k_{SO2} / 10^{-10} cm^3 s^{-1}	k_0 / s^{-1}	[MACRO]$_0$ / 10^{11} cm^{-3}	k_{SO2} / 10^{-10} cm^3 s^{-1}	k_0 / s^{-1}
1	500.4[c]	12.0	2.3[d]	1.8[e]	10.0[f]	1.7	389	9.2	1.8	408
2	500.1	11.9	2.2	1.7	10.4	1.5	393	9.8	1.7	427
3	150.6	10.5	2.8	1.9	8.1	1.0	341	6.6	1.4	332
4	150.4	10.4	2.8	1.9	7.7	1.1	318	6.2	1.8	281

[a] The fitting range is 0.18−10 ms.

[b] "SO_2 scavenge method" means the analysis in which the spectrum at the highest [SO_2] was subtracted from the spectra recorded at lower [SO_2]. The fitting range is 0.38−10 ms.

[c] Balanced by N_2.

[d] The concentration of the precursor is derived by using the cross sections measured in this work, see Supplementary Figure 4.

[e] I_{248nm} represents the photolysis laser fluence.

[f] [MACRO]$_0$ is derived by using the reported peak cross section of 3x10^{-18} cm^2 by Vansco et al.[3]

Supplementary Table 2. Summary of the experimental conditions and fitting results of the reaction of MACRO with H_2O at 298 K.

Exp #	P / Torr	P_{O2} / Torr	[precursor] / 10^{13} cm^{-3}	I_{248nm} / mJ cm^{-2}	$k_{water\text{-}eff}$ / 10^{-17} cm^3 s^{-1}	sd / 10^{-17} cm^3 s^{-1}	k_0 / s^{-1}	# of points
5	501.2[a]	11.8	2.2[b]	1.8[c]	8.0	6.5[d]	370	14
6	150.6	10.5	3.1	1.8	(13.3)[e]		262	10
7	301.0	11.4	3.9	3.6	−1.4		685	2
8	300.4	11.3	6.3	1.7	8.6		578	2
9	150.2	11.4	10.2	1.6	(−5.1)		621	2
10	150.0	11.3	3.7	3.6	(8.9)		469	2
11	500.3	11.8	6.4	1.7	2.6		641	2
12	502.4	12.0	3.0	1.8	−0.3	7.7	563	5
13	500.7	11.9	1.6	1.8	12.9	11.4	334	14
			Weighted average		**8.5**[f]	**4.7**	–	33

[a] Balanced by N_2.

[b] The concentration of the precursor is derived by using the cross sections measured in this work, see Supplementary Figure 4.

[c] I_{248nm} represents the photolysis laser fluence.

[d] *sd* represents the standard deviation of the data.

[e] The data of 150 Torr are shown in the parenthesis and are not included in the final reported value.

[f] The weighted average from six experimental sets (Exp #5,7,8,11,12,13) with the weighting being (# of points – 1).

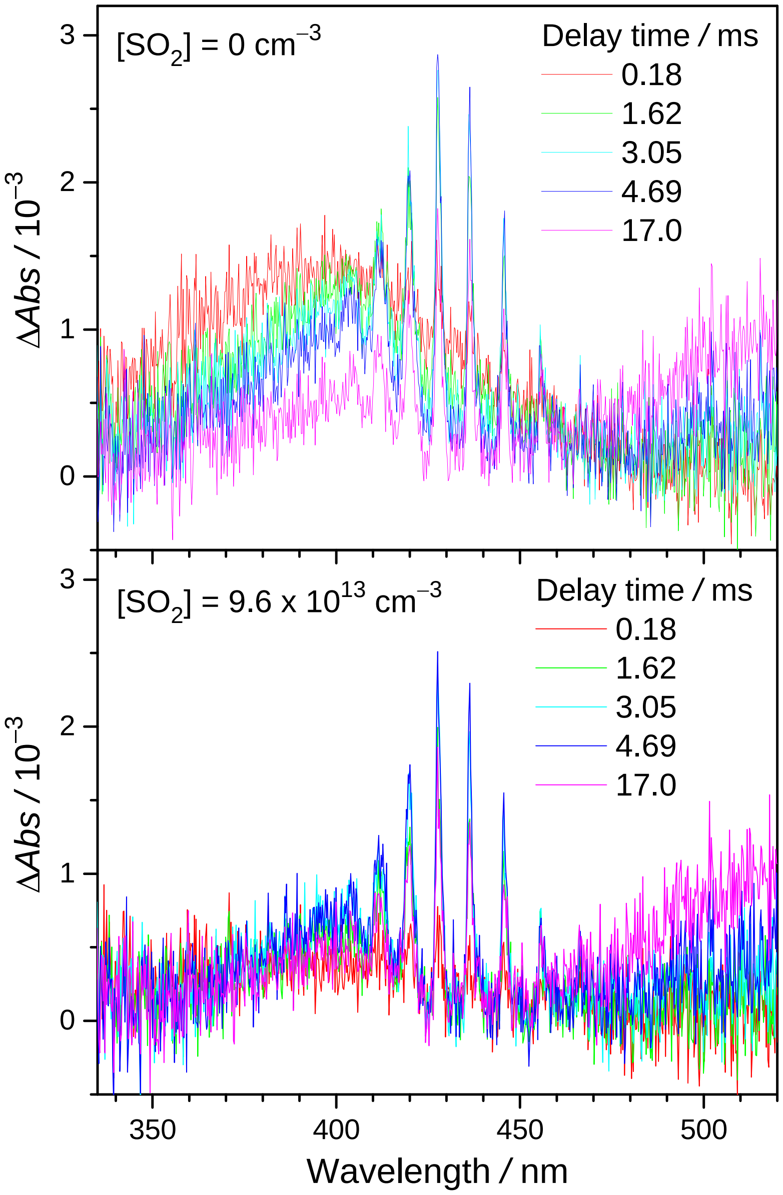

Supplementary Figure 1. Time-resolved difference absorption spectra recorded in the 1,3-diiodo-2- methylprop-1-ene/O_2 photolysis system at 298 K and 500 Torr at different delay times after the photolysis laser pulse. The data are from Exp # 1. Upper panel: The spectra recorded without adding SO_2. Lower panel: The spectra recorded at $[SO_2] = 9.6 \times 10^{13}$ cm^{-3}. Besides MACRO, byproducts IO and I_2 are also observed.

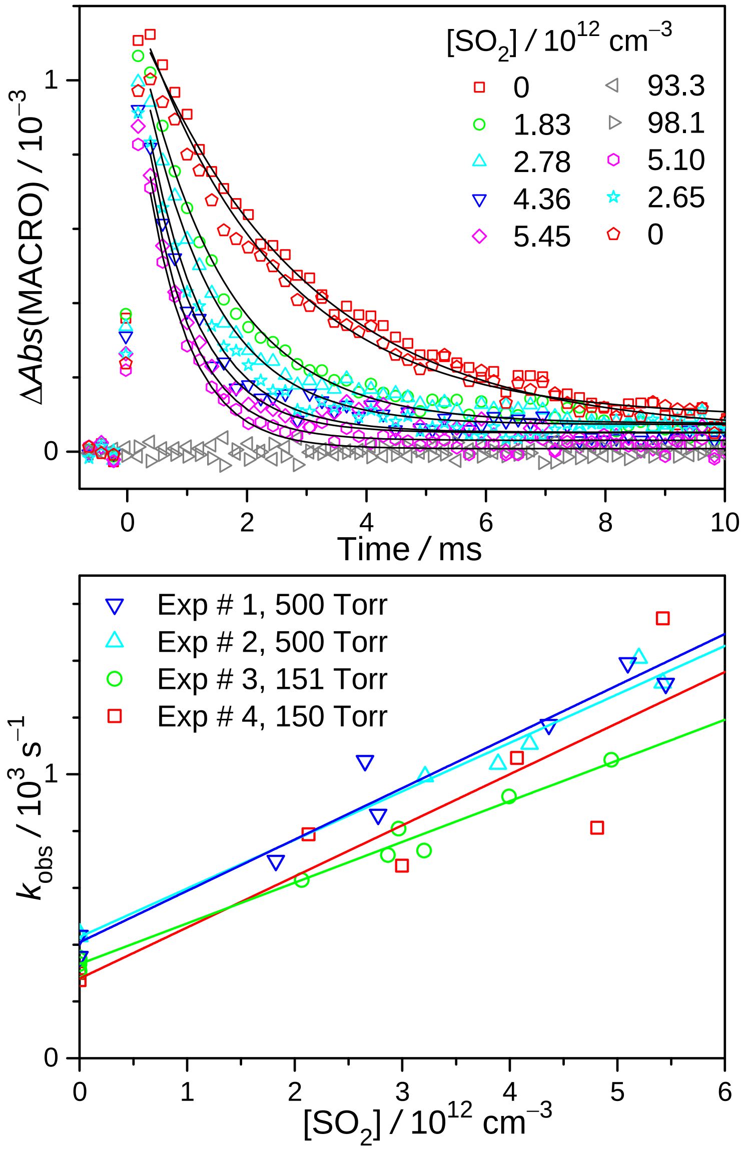

Supplementary Figure 2. Upper panel: MACRO time profiles obtained by fitting the spectra (Exp #1) obtained with the SO_2 scavenge method. The data points ≤ 0.18 ms delay time are not included. Black curves show the fitting results of the exponential function. Lower panel: Pseudo-first-order plot for the reaction of MACRO with SO_2 at 298 K. The data points are from the exponential fitting of the MACRO time profiles.

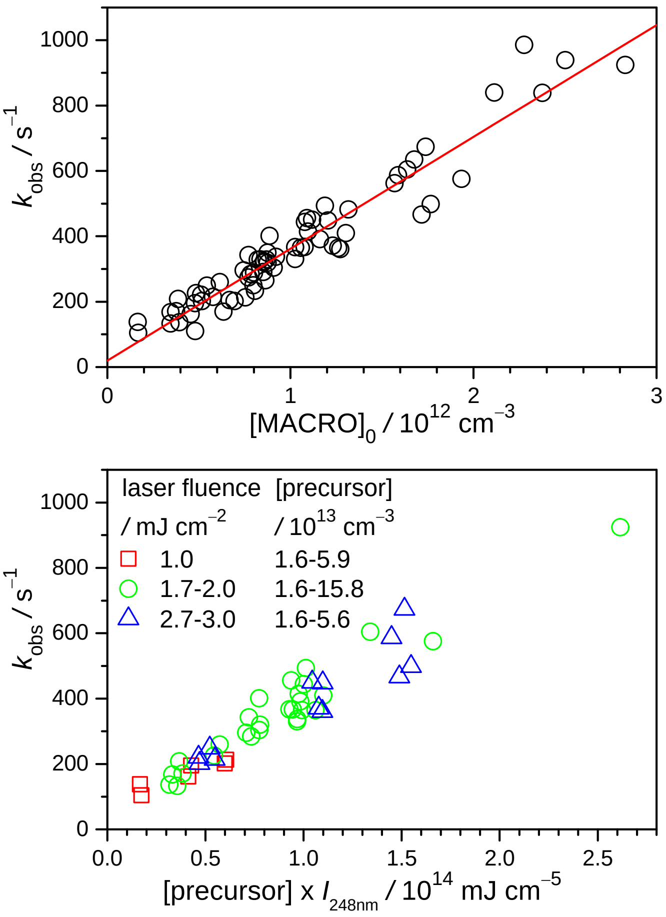

Supplementary Figure 3. Upper: The observed decay rate coefficient k_{obs} plotted against the initial concentration of MACRO, $[MACRO]_0$ (derived by using the peak cross section of $3x10^{-18}$ cm^2 reported by Vansco et al.[3]) when no other reactant was added. Experimental conditions: P = 150–503 Torr; P_{O2} = 5–21 Torr; T = 298 K; [precursor] = (1.6–7.2)x10^{13} cm^{-3} (using the cross sections measured in this work); I_{248nm} = 0.90–3.88 mJ cm^{-2}. The intercept of this plot gives a preliminary estimate of the unimolecular decomposition rate coefficient of MACRO, which is 20±28 s^{-1} (±2 *se*, *se* is the standard error of the intercept). Note that it is very difficult to measure this very small unimolecular decay rate coefficient for *anti*-MACRO, of which the absorption spectrum is very broad and overlaps with that of IO. Here we can only conclude that our experimental results are consistent with the theoretical predictions listed in Table 1. **Lower:** k_{obs} plotted against the product of the initial concentration of the precursor and the photolysis laser fluence, [precursor]xI_{248nm}. A clear linear dependence can be observed in this plot. The data are color-coded for various levels of I_{248nm} and we can see that there is no clear correlation of k_{obs} with I_{248nm} alone. That is, there would also be no clear correlation of k_{obs} with [precursor] alone, since the horizontal axis is their product ([precursor]xI_{248nm}).

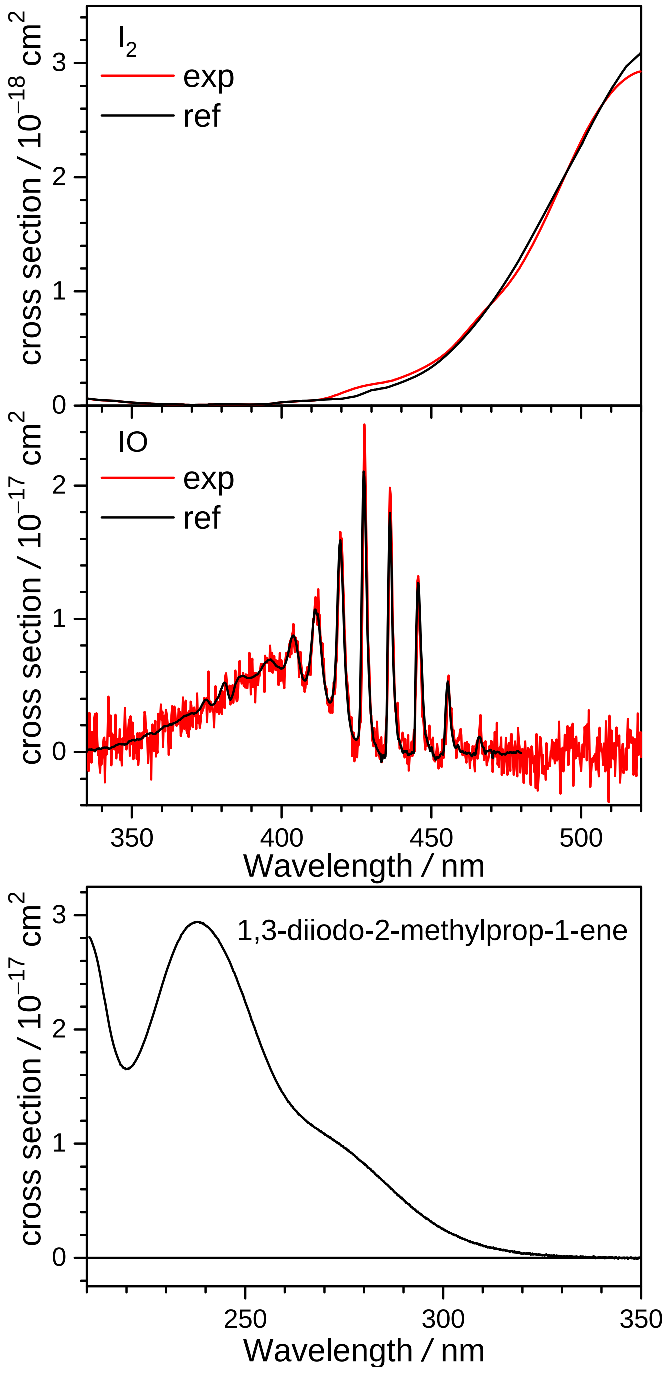

Supplementary Figure 4. The standard I_2 (upper panel) and IO (middle panel) spectra derived from our experimental spectra to include the instrumental functions (shown as red lines). They are consistent with the reported spectra in the literature (black lines).[7,8] The absolute absorption cross sections of the precursor, 1,3-diiodo-2-methylprop-1-ene, measured in this work are shown in the lower panel. The peak cross section was determined to be $(3.0\pm0.2)\text{x}10^{-17}$ cm^2 (±1 *sd*, from 8 measurements) at 238 nm.

We used the *standardized* spectra of MACRO, IO, and I_2 in our analysis to extract their individual contributions. The standard MACRO spectrum is from the Gauss fit of our experimental results (see Figure 3). Supplementary Figure 4 shows the standard spectra of IO and I_2, which are derived from our experimental spectra to include the instrumental functions. This is important for IO, of which the shape of the sharp peaks depends on the resolution of the used spectrometer. We obtained the spectra of IO and I_2 at long photolysis-probe delay times (where the MACRO contribution is gone).

Supplementary Note 2: Alternative simulation for Figure 4 of Newland et al., Atmos. Chem. Phys. 15, 9521–9536 (2015) [Ref. 9].

We estimated the effect of the CI reactions with water vapor in the isoprene ozonolysis system in a manner similar to Newland et al.[9] We separate the stabilized CIs produced in isoprene ozonolysis into two groups, CH_2OO and C4-SCI (MVKO and MACRO). We found that we can fit the results of their Figure 4, but using a very different set of relative yields and rate coefficients (Model 1). The used kinetic parameters of our fit (Model 1) and those used by Newland et al.[9] (Model 2, water-monomer-only scenario) are listed in Supplementary Table 3. See Supplementary Figure 5 for comparison of the simulations.

Supplementary Table 3. Kinetic parameters used to fit the data of Figure 4 of Newland et al.[9] Where k_{H2O}, $k_{(H2O)2}$, and k_{SO2} are the rate coefficients of SCI reactions with water monomer, water dimer, and SO_2, respectively; k_{other} includes the unimolecular decay and other loss processes; ϕ is the relative yield of the SCI in the isoprene ozonolysis.

	SCI	k_{H2O} / cm^3 s^{-1}	$k_{(H2O)2}$ / cm^3 s^{-1}	k_{SO2} / cm^3 s^{-1}	k_{other} / s^{-1}	ϕ
Model 1 (this work)	CH_2OO	3.2×10^{-16} [a]	7.4×10^{-12} [b]	3.9×10^{-11} [c]	15[d]	0.77[d]
	C4-SCI	8×10^{-17} [e]	(1×10^{-14})[f]	3.9×10^{-11} [g]	50[d]	0.23[d]
Model 2[h]	CH_2OO	1.3×10^{-15}	0	3.9×10^{-11}	12	0.54
	C4-SCI	1.1×10^{-15}	0	3.9×10^{-11}	26	0.46

[a] The best known rate coefficient in the literature by Berndt et al.[10]

[b] The best known rate coefficient in the literature by Smith et al.[11]

[c] The widely accepted rate coefficient in the literature by Welz et al.[12]

[d] Fit to the data of Figure 4 of Newland et al.[9]

[e] This work.

[f] This value is too small to be sensitive to the fit.

[g] Assumed the same as that for CH_2OO.

[h] Copied from Table 2 of Newland et al. (water monomer only scenario).[9]

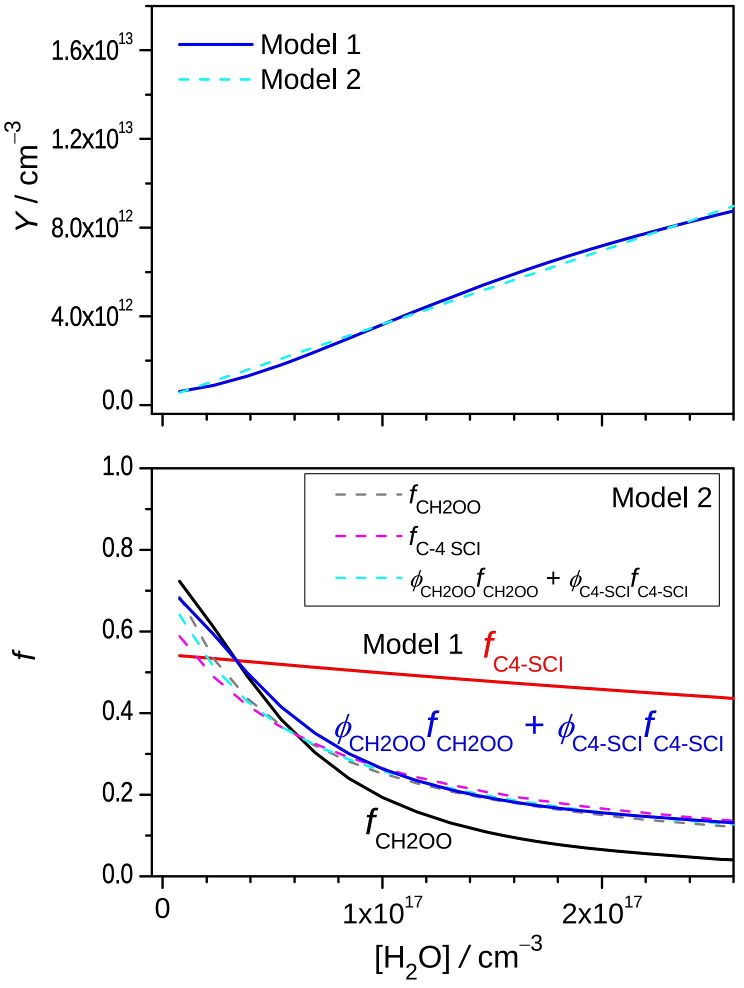

Supplementary Figure 5. Upper: Comparison of the simulation results of Models 1 and 2. Both are fitted to the results of Figure 4 of Newland et al.[9] Lower: Comparison of the *f* values (the fraction of the SCI produced that reacts with SO_2, as defined by Newland et al.[9]) for CH_2OO and C4-SCI and their combined effect in the isoprene ozonolysis. The results of Model 1 are shown as solid lines; those of Model 2 as dashed lines.

This practice indicates that the complicated situation of isoprene ozonolysis can be interpreted with more than one set of kinetic parameters. Note that we do not have full knowledge about the experiments of Newland et al.[9], thus, we should not claim that our fitting can interpret their results. We just like to demonstrate that their situation is complicated and can have more than one interpretation.

We further plot the *f* value (the fraction of the SCI produced that reacts with SO_2, as

defined by Newland et al.[9]) in Supplementary Figure 5 (lower) to illustrate the effects of these two types of CIs. In Model 1, the pronounced decay of f_{CH2OO} with increasing [H_2O] reflects the fact that CH_2OO is quickly consumed by its reactions with water monomer and dimer. However, C4-SCI is consumed by the water reactions much slower. The effect of the remaining CH_2OO in the SCI + SO_2 reactions is insignificant for the range of [H_2O] > 1.5×10^{17} cm^{-3}. As a result, the Y value (as defined in Newland et al.[9]) would not increase in the same way when there is only CH_2OO. In fact, this situation has been proposed by Newland et al. as their possible explanation (iii),[9] which is copied below.

(On page 9529-9530 of Ref. 9) "*(iii) that multiple effects are affecting the curvature of the results shown in Fig. 4. Analogous plots for CH_3CHOO shown in Newland et al. (PCCP 2015) displayed a shallowing gradient with increasing [H_2O] (i.e. the opposite curvature to that caused by the $(H_2O)_2$ reaction). The probable explanation for the curvature observed for CH_3CHOO is the presence of a mix of syn and anti conformers (Scheme 2) in the system and the competing effects of the different kinetics of these two distinct forms of CH_3CHOO. A similar effect may arise for the isoprene derived CRB-SCI which include multiple syn and anti conformers (see Scheme 2). The competition of this effect with that expected from the water dimer reaction may effectively lead to one masking the other under the experimental conditions applied.*"

In addition, the temperature in the experiments of Newland et al. was between 287 and 302 K. This 15 K range may change the rate coefficients by a factor of 2,[11] resulting in extra complications.

Supplementary Note 3: Theoretical Calculations

To obtain the rate coefficients for the unimolecular decomposition of MACRO and the reactions of MACRO with 1 and 2 water molecules, we optimized the reactant and transition state geometries on the singlet ground electronic state using Becke's three parameter hybrid functional (B3LYP)[13,14] with 6-311+G(2d,2p) basis set.[15] Using these geometries, we corrected the electronic energies by quadratic configuration interaction singles, and doubles with perturbative triples (QCISD(T)) method[16] with Dunning's Basis sets[17] extrapolated to complete basis set (CBS) limit.[18] We used the aug-cc-pVXZ (X = D, T, Q) basis sets, and the Hartree−Fock energy was extrapolated using $E_{CBS} + Ae^{-BX}$, and the correlation energy calculated by QCISD(T) was extrapolated using $E_{CBS} + Ae^{-(X-1)} + Be^{-(X-1)^2}$, where X is the cardinal number of the basis set and E_{CBS}, A, and B are optimization parameters. All density functional theory calculations were done with the Gaussian09 program[19] while all QCISD(T) calculations were performed using the MOLPRO program.[20] The rate coefficients were calculated using the conventional transition state theory method using rigid rotor harmonic oscillator approximations using the THERMO program in the Multiwell suite.[21–23] We used the tunneling correction based on the 2nd order vibrational perturbation theory method implemented in the Multiwell suite.[24] This method was used previously for C1 to C3 CIs and has shown good predictability.[25,26]

Supplementary Figure 6 shows the optimized geometries and relevant energies for the MACRO conformers. Our results are very similar to those of the previous works by Vereecken et al.[27] and Vansco et al.[3]

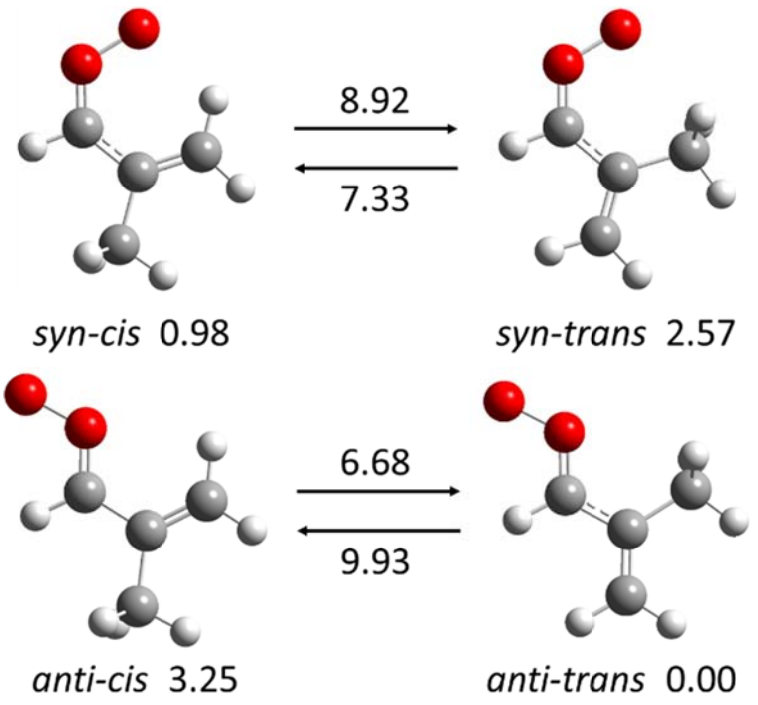

Supplementary Figure 6. Optimized structures of the four conformers of MACRO. The zero-point-corrected energies (in kcal mol^{-1}) from this work are shown below each structure. Energies are calculated using the electronic energies given at QCISD(T)/CBS// B3LYP/6-311+G(2d,2p) and harmonic zero-point correction at B3LYP/6-311+G(2d,2p). The double arrows indicate that the barrier between the *trans* and *cis* conformers within each *syn* or *anti* family is low, allowing rapid interconversion at room temperature, with the forward and backward barrier energies shown above and below the arrows.

For calculating the rate coefficients of the reactions of Criegee intermediate with water monomer and dimer, it is important to calculate the reaction barrier energies using high level quantum chemistry methods including complete basis set extrapolation. However, due to the limitation of our computational resource, we were not able to perform the QCISD(T)/aug-cc-pVQZ calculation for the MACRO+$(H_2O)_2$ reaction, which is required to obtain the CBS energy. On the other hand, we noticed there is a strong correlation between the H_2O versus $(H_2O)_2$ reactions in the differences between the QCISD(T)/aug-cc-pVTZ and QCISD(T)/CBS energies for the alkyl substituted C1 to C3 CIs calculated previously.[26] The difference of the reaction barrier (from the free reactants to the transition state, $E_b = E(\text{TS}) - E(\text{free reactants})$) calculated at aug-cc-pVTZ (AVTZ) and CBS levels for the water monomer reaction and the water dimer reaction shows a linear relation for the alkyl Criegee intermediates as shown in Supplementary Figure 7 and Supplementary Table 4.

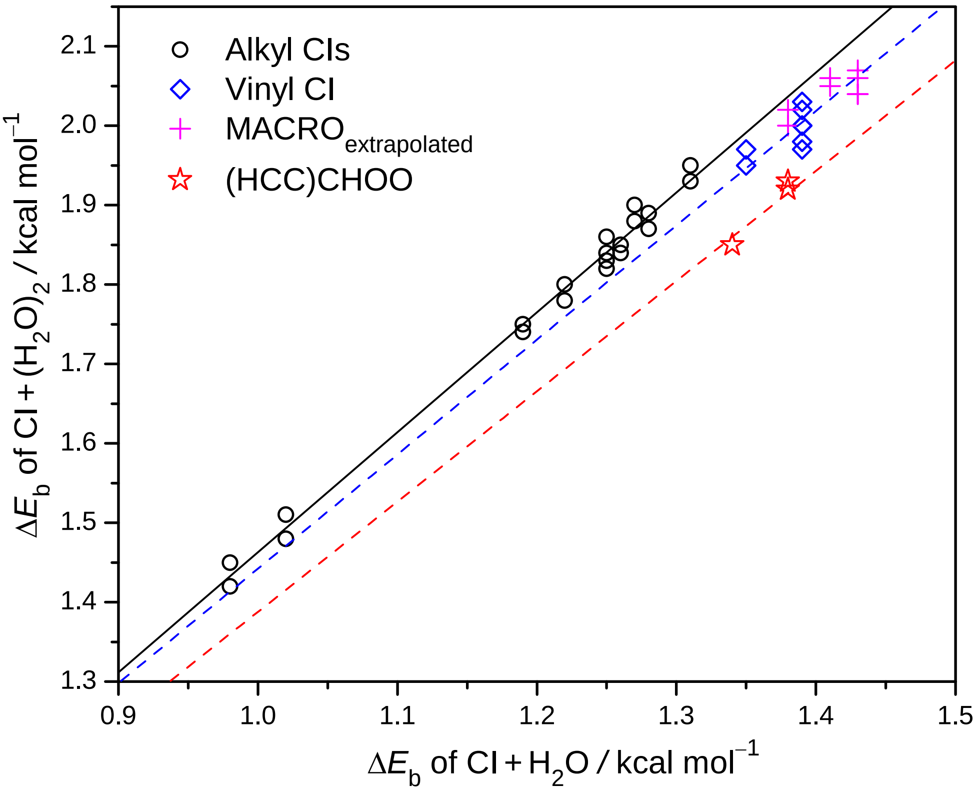

Supplementary Figure 7. Horizontal coordinate: energy difference between QCISD(T)/CBS and QCISD(T)/AVTZ, $\Delta E_b = E_b(\text{CBS}) - E_b(\text{AVTZ})$, for the transition state of CI+H_2O reaction; Vertical coordinate: ΔE_b for the transition state of CI+$(H_2O)_2$ reaction. The black solid line is a linear fit to all the alkyl CI data points (listed in Supplementary Table 4). The dashed blue and red lines are through the origin and fitted to the data of the vinyl CI and (HC≡C)CHOO, respectively.

The pairing used to plot Supplementary Figure 7 and calculate the extrapolated CBS values is based on the orientation of the free OH bond of the H_2O molecule that is bonded to the carbonyl carbon of the CI, as shown in Supplementary Figure 8. The 1a channel in the water monomer reaction is paired with the 2b and 2c channels in the water dimer reaction, while the 1b channel is paired with the 2a and 2d channels.

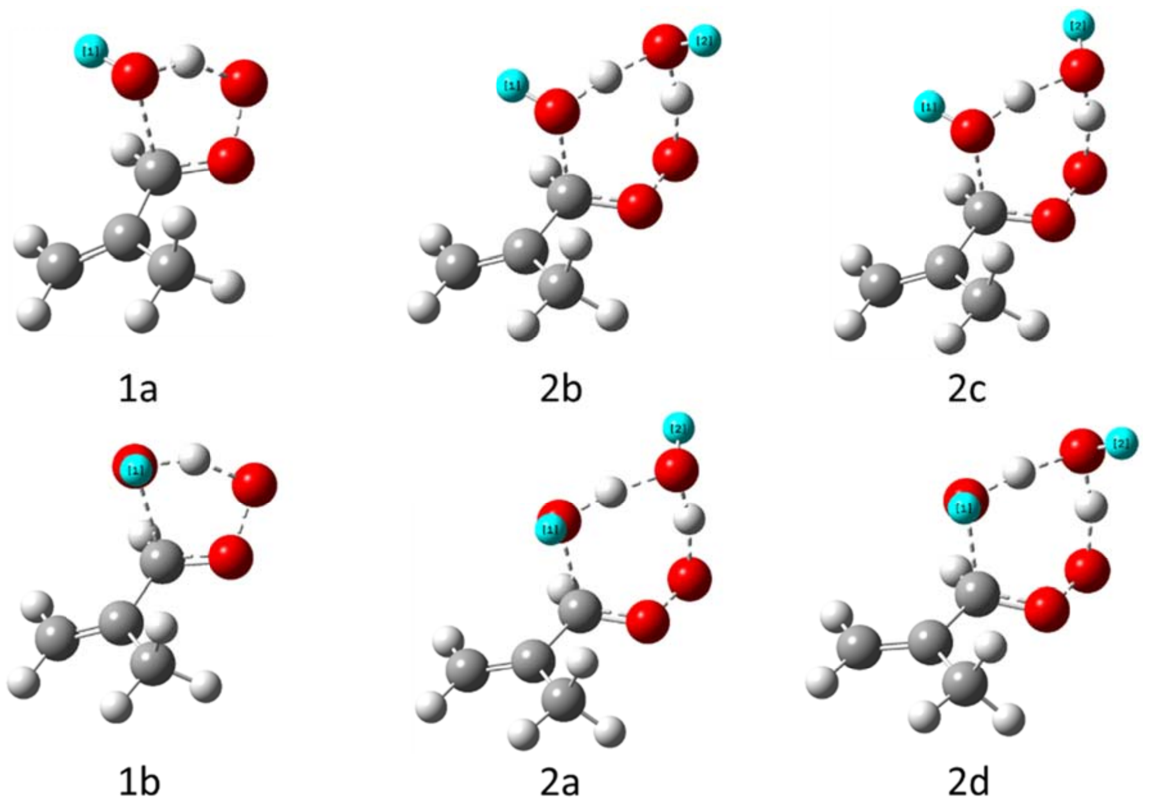

Supplementary Figure 8. Optimized transition state structures of the reaction channels of *anti-trans* MACRO; 1a and 1b are for the water monomer reactions and 2a, 2b, 2c, and 2d for the water dimer reactions.

We also noticed the similarity in geometry between *anti*-(CH_2=CH)CHOO and *anti*-MACRO. The only difference is that *anti*-MACRO has a CH_3 group, which is mainly a spectator in the reaction with water molecule(s). The QCISD(T)/CBS reaction barrier heights (transition state energy relative to the free reactants) of *anti*-(CH_2=CH)CHOO+H_2O and *anti*-MACRO+H_2O are also very close to each other (see Supplementary Table 4), further confirming the idea that we can estimate the CBS value of *anti*-MACRO+$(H_2O)_2$ by the following strategy:

(i) We used B3LYP geometries to perform single point calculation with QCISD(T)/AVTZ for all the stationary points of *anti*-CH_2CHCHOO, *anti*-MACRO, H_2O, $(H_2O)_2$, *anti*-CH_2CHCHOO+H_2O, *anti*-CH_2CHCHOO+$(H_2O)_2$, *anti*-MACRO+H_2O, and *anti*-MACRO+$(H_2O)_2$.

(ii) For the stationary points of *anti*-CH_2CHCHOO, *anti*-MACRO, H_2O, $(H_2O)_2$, *anti*-CH_2CHCHOO+H_2O, *anti*-CH_2CHCHOO+$(H_2O)_2$, and *anti*-MACRO+H_2O, we performed QCISD(T)/aug-cc-pVQZ calculations and obtained the CBS energy using the

equation given above.

(iii) We obtained the energy difference between the QCISD(T)/CBS and QCISD(T)/aug-cc-pVTZ energies, $\Delta E_b = E_b(\text{CBS}) - E_b(\text{AVTZ})$, for the reaction barrier for *anti*-$CH_2CHCHOO$+H_2O, *anti*-$CH_2CHCHOO$+$(H_2O)_2$, and *anti*-MACRO+H_2O. Then, the approximate reaction barrier of CBS energy of the MACRO+$(H_2O)_2$ is obtained by the following equation and plotted in Supplementary Figure 7.

$$\Delta E_b^i(\text{MACRO}) = \frac{\Delta E_b^i(\text{vinyl CI})}{\Delta E_b^j(\text{vinyl CI})}\Delta E_b^j(\text{MACRO})$$

When i = 2a or 2d, j = 1b; when i = 2b or 2c, j = 1a. Similar strategy was used to estimate the barriers for the reaction involving *syn*-MACRO by using the results of *syn*-$CH_2CHCHOO$.

Supplementary Table 4. Zero-point corrected energies for the transition state, in kcal mol^{-1}, relative to the free reactants. Harmonic zero-point correction are from B3LYP/6-311+G(2d,2p). Electronic energies are from QCISD(T)/AVTZ//B3LYP/6-311+G(2d,2p) and QCISD(T)/CBS// B3LYP/6-311+G(2d,2p). We also listed the difference between CBS and AVTZ barrier energies, which is used to plot Supplementary Figure 7. The values with parentheses are estimated by the extrapolation method mentioned above.

Criegee intermediates	H_2O	Eb(AVTZ)	Eb(CBS)	ΔE_b	$(H_2O)_2$	Eb(AVTZ)	Eb(CBS)	ΔE_b
CH_2OO	1a	2.67	3.69	1.02	2a	-8.03	-6.60	1.42
	1b	1.83	2.81	0.98	2b	-8.00	-6.51	1.48
					2c	-6.85	-5.34	1.51
					2d	-7.84	-6.39	1.45
anti	1a	-0.25	0.97	1.22	2a	-9.19	-7.45	1.74
CH_3CHOO	1b	-0.84	0.35	1.19	2b	-9.50	-7.72	1.78
					2c	-8.30	-6.51	1.80
					2d	-8.96	-7.20	1.75
anti_Syn	1a	-0.91	0.40	1.31	2a	-9.49	-7.61	1.87
CH_3CH_2CHOO	1b	-1.27	0.01	1.28	2b	-10.07	-8.14	1.93
					2c	-8.92	-6.97	1.95
					2d	-9.21	-7.32	1.89
anti_Gauche	1a	-0.65	0.62	1.27	2a	-9.30	-7.47	1.84

CH_3CH_2CHOO	1b	-1.04	0.21	1.25	2b	-9.96	-8.08	1.88
					2c	-8.78	-6.88	1.90
					2d	-9.09	-7.24	1.86
	1a'	-0.35	0.90	1.26	2a'	-9.76	-7.94	1.82
	1b'	-1.02	0.23	1.25	2b'	-9.98	-8.14	1.84
					2c'	-8.78	-6.93	1.85
					2d'	-9.53	-7.70	1.83
anti_syn	1a	2.54	3.92	1.39	2a	-6.69	-4.74	1.95
$CH_2CHCHOO$	1b	1.57	2.92	1.35	2b	-5.89	-3.88	2.02
(vinyl CI)					2c	-4.59	-2.56	2.03
					2d	-6.47	-4.50	1.97
anti_anti	1a	3.59	4.98	1.39	2a	-4.70	-2.73	1.97
$CH_2CHCHOO$	1b	2.55	3.94	1.39	2b	-4.67	-2.67	2.00
(vinyl CI)					2c	-3.39	-1.39	2.00
					2d	-4.42	-2.44	1.98
anti	1a	4.41	5.79	1.38	2a	-4.91	-3.06	1.85
CHCCHOO	1b	3.19	4.53	1.34	2b	-4.25	-2.33	1.92
					2c	-2.95	-1.02	1.93
					2d	-4.65	-2.80	1.85
anti-cis	1a	1.84	3.25	1.41	2a	-7.21	(-5.21)	(2.00)
MACRO	1b	0.36	1.75	1.38	2b	-6.56	(-4.51)	(2.05)
					2c	-5.47	(-3.41)	(2.06)
					2d	-7.19	(-5.17)	(2.02)
anti-trans	1a	2.75	4.18	1.43	2a	-4.75	(-2.71)	(2.04)
MACRO	1b	2.36	3.79	1.43	2b	-5.32	(-3.26)	(2.07)
					2c	-4.28	(-2.22)	(2.06)
					2d	-4.38	(-2.33)	(2.04)
syn_syn	1a	9.58	10.95	1.37	2a	-0.39	1.67	2.06
$CH_2CHCHOO$	1b	9.18	10.55	1.37	2b	-0.64	1.38	2.02
(vinyl CI)					2c	0.69	2.70	2.01
					2d	-0.23	1.90	2.13
syn_anti	1a	6.60	7.97	1.37	2a	-2.06	0.02	2.08
$CH_2CHCHOO$	1b	6.59	7.99	1.40	2b	-3.47	-1.47	2.01

(vinyl CI)					2c	-2.04	-0.04	2.00
					2d	-2.21	-0.08	2.14
syn-cis	1a	8.46	9.87	1.41	2a	-1.47	(0.66)	(2.13)
MACRO	1b	7.49	8.91	1.42	2b	-1.14	(0.95)	(2.08)
					2c	-0.03	(2.04)	(2.07)
					2d	-1.35	(0.85)	(2.20)
syn-trans	1a	5.30	6.69	1.39	2a	-2.88	(-0.79)	(2.09)
MACRO	1b	5.20	6.61	1.41	2b	-4.11	(-2.09)	(2.03)
					2c	-2.30	(-0.29)	(2.01)
					2d	-3.11	(-0.96)	(2.15)

Supplementary Table 5. Theoretical rate coefficients for MACRO+H_2O and MACRO+$(H_2O)_2$. The last two columns are the total water monomer and dimer reaction rate coefficients estimated by the Boltzmann distributions of the fast-interconverting *cis* and *trans* conformers.

Temp (K)	*anti-trans-* MACRO + H_2O (10^{-17} cm^3 s^{-1})	*anti-trans-* MACRO + $(H_2O)_2$ (10^{-14} cm^3 s^{-1})	*anti-cis-* MACRO + H_2O (10^{-15} cm^3 s^{-1})	*anti-cis-* MACRO + $(H_2O)_2$ (10^{-13} cm^3 s^{-1})	*anti-* MACRO + H_2O (10^{-17} cm^3 s^{-1})	*anti-* MACRO + $(H_2O)_2$ (10^{-14} cm^3 s^{-1})
273.15	2.78	3.99	1.27	5.45	3.10	4.12
278.15	3.05	3.25	1.30	4.32	3.41	3.36
283.15	3.35	2.68	1.34	3.47	3.75	2.78
288.15	3.66	2.22	1.37	2.79	4.12	2.30
293.15	3.99	1.85	1.41	2.27	4.50	1.93
**298.15**	**4.34**	**1.55**	**1.45**	**1.86**	**4.92**	**1.62**
303.15	4.71	1.31	1.49	1.53	5.36	1.37
308.15	5.10	1.11	1.54	1.27	5.83	1.17

Temp (K)	*syn-trans-* MACRO + H_2O (10^{-19} cm^3 s^{-1})	*syn-trans-* MACRO + $(H_2O)_2$ (10^{-15} cm^3 s^{-1})	*syn-cis-* MACRO + H_2O (10^{-20} cm^3 s^{-1})	*syn-cis-* MACRO + $(H_2O)_2$ (10^{-17} cm^3 s^{-1})	*syn-* MACRO + H_2O (10^{-20} cm^3 s^{-1})	*syn-* MACRO + $(H_2O)_2$ (10^{-17} cm^3 s^{-1})
273.15	3.87	1.81	0.86	4.89	0.96	5.33
278.15	4.05	1.63	0.86	4.55	0.97	4.99
283.15	4.37	1.47	0.91	4.27	1.05	4.71
288.15	4.69	1.33	0.96	4.01	1.12	4.45
293.15	5.20	1.21	1.05	3.80	1.24	4.24
**298.15**	**5.78**	**1.10**	**1.18**	**3.62**	**1.41**	**4.06**
303.15	6.41	1.01	1.31	3.44	1.59	3.88
308.15	7.23	0.93	1.51	3.30	1.86	3.74

Supplementary Table 6. Theoretical rate coefficients of the unimolecular reactions for MACRO conformers. The last two columns are the total reaction rate coefficients estimated by the Boltzmann distributions of the fast-interconverting *cis* and *trans* conformers.

Temp (K)	*anti-trans-* MACRO (s^{-1})	*anti-cis-* MACRO (s^{-1})	*syn-trans-* MACRO (s^{-1})	*syn-cis-* MACRO (s^{-1})	*anti-* MACRO (s^{-1})	*syn-* MACRO (s^{-1})
273.15	2	104	0	398	2	378
278.15	3	166	1	605	3	573
283.15	5	260	1	908	6	857
288.15	8	401	2	1342	10	1264
293.15	13	610	4	1959	16	1839
**298.15**	**21**	**916**	**6**	**2824**	**25**	**2644**
303.15	34	1356	9	4022	40	3755
308.15	52	1983	15	5666	62	5274

We also comment on using the E_b values of the water monomer reactions to estimate the E_b values of the water dimer reactions, which was used in previous studies.[27] As given in Supplementary Figure 9, we can see a correlation between them. However, one important aspect is that the trend for the simplest CI CH_2OO (black dashed line) is quite different from the correlation involving unsaturated CIs (red dashed line). In addition, the deviations from the linear fit (Supplementary Figure 9) are much larger than those in the case when we use ΔE_b (Supplementary Figure 7). Therefore, we think it is more efficient and accurate to use the ΔE_b strategy to approximate the QCISD(T)/CBS energies for the MACRO+$(H_2O)_2$ reaction.

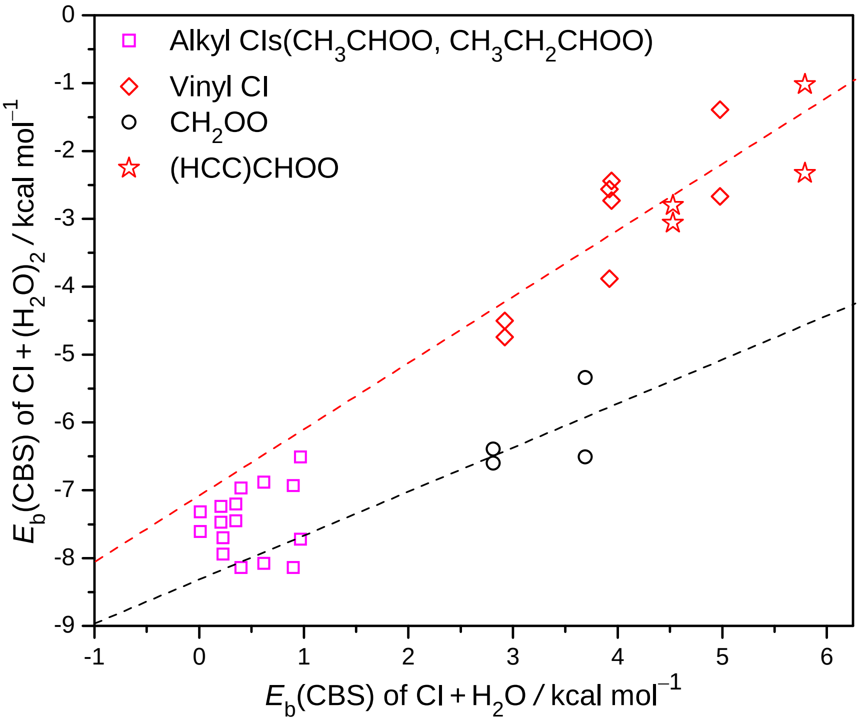

Supplementary Figure 9. Horizontal coordinate: QCISD(T)/CBS E_b for the transition state of CI+H_2O reaction; Vertical coordinate: QCISD(T)/CBS E_b for the transition state of CI+$(H_2O)_2$ reaction. The dashed black and red lines are fitted to the data of the simplest CI CH_2OO and unsaturated CIs ((HC≡C)CHOO, (CH_2=CH)CHOO), respectively.

Justification for the theoretical methods

Concerning the accuracy of the present QCISD(T)//B3LYP method, we have confirmed that the QCISD(T) gives energies that are consistent with CCSD(T) and multireference methods for the CH_2OO+H_2O reaction (difference ~ 1 kcal mol^{-1}, see Table S3 of Lin et al.[28]). Part of the table is reproduced below for reference.

Supplementary Table 7. Zero-point corrected energies, in kcal mol^{-1}, for the CH_2OO+H_2O 1b TS calculated by multireference and single reference methods with aug-cc-pVTZ. The geometry and zero-point correction were calculated with B3LYP/6-311+(2d,2p), and the zero of energy is the VDW complex $CH_2OO\cdots H_2O$. Adapted from Lin et al.[28]

Method	*E*(TS) – *E*(VDW)
QCISD(T)	8.64
CCSD(T)	8.39
MRCI+Q Davidson (10,10)	9.79
MRCI+Q Pople (10,10)	8.96
CASPT2 (10,10)	8.52

In addition, we have confirmed that the B3LYP geometries of the TS for the CH_2OO+H_2O and $CH_2OO+(H_2O)_2$ reactions are similar to the QCISD(T) geometries (Table S5 of Lin et al. PCCP, 18, 4557, 2016) and that this difference in geometry by the 2 quantum chemistry methods can induce an error of 0.3 kcal mol^{-1} in the TS energies. Furthermore, the harmonic frequencies by the B3LYP and QCISD(T) for the CH_2OO+H_2O TS differ by 20 cm^{-1} at most for the low frequency modes (<1000 cm^{-1}). This difference in harmonic frequencies can give a variation of 0.08 kcal mol^{-1} in the free energy at 298 K, which is smaller than the variation in the quantum chemistry energies.

For MACRO, the harmonic approximation may not be valid for the CH_3 internal-rotation mode. Therefore, we have calculated the potential energy along the CH_3 torsional angle for MACRO and the TS of MACRO+H_2O 1b reaction. We find that the torsional potential energy curves at both geometries are nearly identical, and thereby the free energy correction for this mode will also cancel out when we calculate the free energy difference.

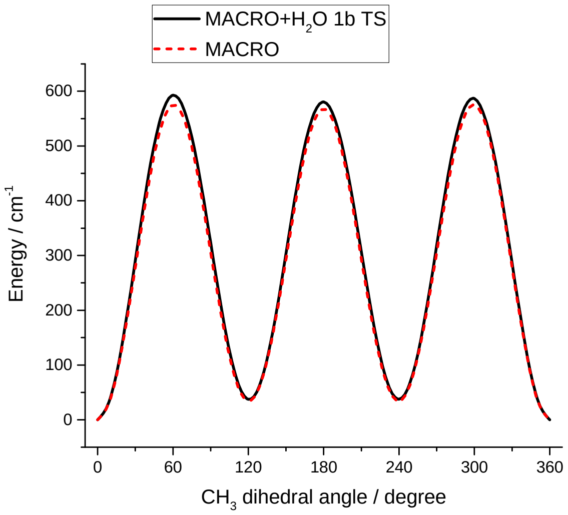

Supplementary Figure 10. Potential energy curves along the CH_3 torsional dihedral angle for MACRO and the TS of MACRO+H_2O 1b reaction calculated using B3LYP/6-311+G(2d,2p).

Error of the theoretical k_{uni}

Electronic energy. Concerning the error of the rate coefficient of the unimolecular reaction, similar to the reaction with water, we can assign the main error source to the uncertainties in the electronic energies obtained by QCISD(T). Here we refer to our previous paper by Yin and Takahashi[25] concerning the theoretical calculation of unimolecular decomposition rates for different Criegee intermediates. For *anti*-MACRO, the unimolecular reaction proceeds through the OO bending channel forming dioxirane[3,27,29], similar to that of CH_2OO.[25,30] In **Supplementary Table 8**, we summarized the barrier energies of the OO bending channel for CH_2OO calculated at various theoretical methods. Compared to the barrier energies calculated by multireference methods, QCISD(T) can give an underestimation of 1.2 kcal mol^{-1}. On the other hand, the core correlation effect is minor but can result in a variation of 0.2 kcal mol^{-1}. Furthermore, for *anti-trans*-MACRO, we found that QCISD(T)/aug-cc-pVTZ gives a barrier energy that is 0.4 kcal mol^{-1} smaller than that obtained with CCSD(T)/aug-cc-pVTZ. This trend is similar to that for CH_2OO. Therefore, we can expect a similar underestimation for the barrier energy of the *anti-trans*-MACRO unimolecular reaction.

Supplementary Table 8. Zero-point corrected energies, in kcal mol^{-1}, for the barrier energy of the CH_2OO unimolecular decomposition to form dioxirane calculated by multireference and single reference methods with complete basis set extrapolation (CBS). The geometry and zero-point correction were calculated with B3LYP/6-311+(2d,2p), and the zero of energy is CH_2OO at equilibrium geometry. Adapted from Yin and Takahashi.[25]

Method	Barrier energy
QCISD(T)	18.71
QCISD(T) with core correlation	18.99
CCSD(T)	19.33
MRCI+Q Davidson (10,10)	19.90

Hindered rotor partition function (free energy). We have calculated the free energy by directly counting the eigenstates for the methyl-rotor Schrodinger equation obtained from the potential energy curve calculated using B3LYP/6-311+(2d,2p), given in Figure S10. Compared to the harmonic approximation, the one obtained from the direct counting is smaller by 0.04 kcal mol^{-1}, which is negligible compared to the error in the electronic energy.

Tunneling correction.

In the present calculation of the unimolecular rate coefficient for MACRO, we have included the tunneling correction, but as shown below, the effect is minor. At room temperature, it will only lead to a correction of 1.2 times for the most stable *anti-trans* conformer.

Supplementary Table 9. Tunneling effect of the dominant unimolecular reaction of MACRO calculated by the vibrational 2nd order perturbation method. For the first three species, the dominant unimolecular reaction is OO bending reaction; for *syn-cis*, it is CCCOO ring closure reaction.

T / K	*anti-trans*	*anti-cis*	*syn-trans*	*syn-cis*
273.15	1.27	1.26	1.50	1.38
278.15	1.26	1.25	1.47	1.36
283.15	1.25	1.24	1.45	1.35
288.15	1.24	1.23	1.43	1.33
293.15	1.23	1.22	1.41	1.32
298.15	1.22	1.21	1.40	1.31
303.15	1.21	1.20	1.38	1.30
308.15	1.21	1.20	1.36	1.29

Summary. Our analysis indicates that the partition function calculation and tunneling correction have minor effects and that the currently used QCISD(T)/CBS method underestimates the reaction barrier height and thus overestimates the reaction rate coefficient. Therefore, our theoretical number in Table 1 would represent an upper limit for the unimolecular reaction rate coefficient.

References

1. Chao, W., Hsieh, J., Chang, C. & Lin, J. J. Direct kinetic measurement of the reaction of the simplest Criegee intermediate with water vapor. *Science* **347**, 751–754 (2015).
2. Lin, Y. H., Li, Y. L., Chao, W., Takahashi, K. & Lin, J. J. M. The role of the iodine-atom adduct in the synthesis and kinetics of methyl vinyl ketone oxide - A resonance-stabilized Criegee intermediate. *Phys. Chem. Chem. Phys.* **22**, 13603–13612 (2020).
3. Vansco, M. F. *et al.* Synthesis, Electronic Spectroscopy, and Photochemistry of Methacrolein Oxide: A Four-Carbon Unsaturated Criegee Intermediate from Isoprene Ozonolysis. *J. Am. Chem. Soc.* **141**, 15058–15069 (2019).
4. Manatt, S. L. & Lane, A. L. A compilation of the absorption cross-sections of SO_2 from 106 to 403 nm. *J. Quant. Spectrosc. Radiat. Transf.* **50**, 267–276 (1993).
5. Kuo, M. T., Takahashi, K. & Lin, J. J. M. Reactions of Criegee Intermediates are Enhanced by Hydrogen-Atom Relay Through Molecular Design. *ChemPhysChem* **21**, 2056–2059 (2020).
6. Li, Y.-J., Lin, C.-Y., Lin, Y.-H. & Lin, J. J.-M. Temperature-dependent kinetics of the simplest Criegee Intermediate reaction with dimethyl sulfoxide. *J. Chinese Chem. Soc.* **67**, 1563–1570 (2020).
7. Sander, S. P. *et al.* Chemical Kinetics and Photochemical Data for Use in Atmospheric Studies, Evaluation No. 17. *JPL Publ. 10-6* **Jet Propul**, (2011).
8. Spietz, P., Gómez Martín, J. C. & Burrows, J. P. Spectroscopic studies of the I_2/O_3 photochemistry Part 2. Improved spectra of iodine oxides and analysis of the IO absorption spectrum. *J. Photochem. Photobiol. A Chem.* **176**, 50–67 (2005).
9. Newland, M. J. *et al.* Atmospheric isoprene ozonolysis: impacts of stabilised Criegee intermediate reactions with SO_2, H_2O and dimethyl sulfide. *Atmos. Chem. Phys.* **15**, 9521–9536 (2015).

10. Berndt, T. *et al.* Kinetics of the unimolecular reaction of CH_2OO and the bimolecular reactions with the water monomer, acetaldehyde and acetone at atmospheric conditions. *Phys. Chem. Chem. Phys.* **17**, 19862–19873 (2015).

11. Smith, M. C. *et al.* Strong Negative Temperature Dependence of the Simplest Criegee Intermediate CH_2OO Reaction with Water Dimer. *J. Phys. Chem. Lett.* **6**, 2708–2713 (2015).

12. Weltz, O. *et al.* Direct Kinetic Measurement of Ciregee Intermediat (CH_2OO) Formed by Reaction of CH_2I with O_2. *Science* **335**, 204–207 (2012).

13. Becke, A. Density Functional Thermochemistry III The Role of Exact Exchange. *J. Chem. Phys.* **98**, 5648–5652 (1993).

14. Lee, C., Yang, W. & Parr, R. G. Development of the Colle-Salvetti correlation-energy formula into a functional of the electron density. *Phys. Rev. B* **37**, 785–789 (1988).

15. Krishnan, R., Binkley, J. S., Seeger, R. & Pople, J. A. Self-consistent molecular orbital methods. XX. A basis set for correlated wave functions. *J. Chem. Phys.* **72**, 650–654 (1980).

16. Pople, J. A., Head-Gordon, M. & Raghavachari, K. Quadratic configuration interaction . A general technique for determining electron correlation energies. *J. Chem. Phys.* **87**, 5968–5975 (1987).

17. Dunning, T. H. Gaussian basis sets for use in correlated molecular calculations. I. The atoms boron through neon and hydrogen. *J. Chem. Phys.* **90**, 1007–1023 (1989).

18. Peterson, K. A., Woon, D. E. & Dunning, T. H. Benchmark calculations with correlated molecular wave functions. IV. The classical barrier height of the $H+H_2 \rightarrow H_2+H$ reaction. *J. Chem. Phys.* **100**, 7410–7415 (1994).

19. Frisch, M. J. *et al.* Gaussian09 A.02. (2009).

20. Werner, H.-J. *et al.* MOLPRO Ver 2010.1. (2011).

21. Barker, J. R. *et al.* MultiWell. *MultiWell-2016* (2016).

22. Barker, J. R. Reaction Systems . I . MultiWell Computer Program Suite. *Int. J. Chem. Kinet.* **33**, 232–245 (2000).

23. Barker, J. R. Energy Transfer in Master Equation Simulations: A New Approach. *Int. J. Chem. Kinet.* **41**, 748–763 (2009).

24. Miller, W. H., Hernandez, R., Handy, N. C., Jayatilaka, D. & Willetts, A. Ab initio calculation of anharmonic constants for a transition state, with application to semiclassical transition state tunneling probabilities. *Chem. Phys. Lett.* **172**, 62–68 (1990).

25. Yin, C. & Takahashi, K. How does substitution affect the unimolecular reaction rates of Criegee intermediates? *Phys. Chem. Chem. Phys.* **19**, 12075–12084 (2017).

26. Yin, C. & Takahashi, K. Effect of unsaturated substituents in the reaction of Criegee intermediates with water vapor. *Phys. Chem. Chem. Phys.* **20**, 20217–20227 (2018).

27. Vereecken, L., Novelli, A. & Taraborrelli, D. Unimolecular decay strongly limits the atmospheric impact of Criegee intermediates. *Phys. Chem. Chem. Phys.* **19**, 31599–31612 (2017).

28. Lin, L.-C. *et al.* Competition between H_2O and $(H_2O)_2$ reactions with CH_2OO/CH_3CHOO. *Phys. Chem. Chem. Phys.* **18**, 4557–4568 (2016).

29. Kuwata, K. T. & Valin, L. C. Quantum chemical and RRKM/master equation studies of isoprene ozonolysis: Methacrolein and methacrolein oxide. *Chem. Phys. Lett.* **451**, 186–191 (2008).

30. Nguyen, T. L., Lee, H., Matthews, D. A., McCarthy, M. C. & Stanton, J. F. Stabilization of the Simplest Criegee Intermediate from the Reaction between Ozone and Ethylene: A High-Level Quantum Chemical and Kinetic Analysis of Ozonolysis. *J. Phys. Chem. A* **119**, 5524–5533 (2015).
