# Supplementary material for: Surprisingly long lifetime of methacrolein oxide, an isoprene derived Criegee intermediate, under humid conditions
Source: Commun Chem. 2021 Feb 5;4:12. doi: 10.1038/s42004-021-00451-z (PMC9814537; doi:10.1038/s42004-021-00451-z)
Supplement: Supplementary file 2 — Description of Additional Supplementary Files [file 42004_2021_451_MOESM2_ESM.pdf]

Description of Additional Supplementary Files

File Name: Supplementary Data 1
Description: This file (Supplementary Data 1.xlsx) contains the optimized XYZ geometries for the MACRO, pre-reactive complex (PC), transition state (TS), and product (Prod), obtained at B3LYP/6-311+G(2d,2p). Unit: Angstrom.
